# Disease burden and attributable risk factors of neonatal disorders and their specific causes in China from 1990 to 2019 and its prediction to 2024

**DOI:** 10.1186/s12889-023-15050-x

**Published:** 2023-01-18

**Authors:** Yuhang Wu, Fan Xia, Mengshi Chen, Senmao Zhang, Ziqi Yang, Ziqiang Gong, Xuan Zhou, Lizhang Chen, Tingting Wang

**Affiliations:** 1 Department of Epidemiology and Health Statistics, Xiangya School of Public Health, Central South University, Changsha, China; 2 Hunan Provincial Key Laboratory of Clinical Epidemiology, Xiangya School of Public Health, Central South University, Changsha, 410008 China; 3 NHC Key Laboratory of Birth Defect for Research and Prevention, Hunan Provincial Maternal and Child Health Care Hospital, Changsha, Hunan China

**Keywords:** Neonatal disorders, Disease burden, Prediction, Risk factors, China

## Abstract

**Background:**

Neonatal health is a cornerstone for the healthy development of the next generation and a driving force for the progress of population and society in the future. Updated information on the burden of neonatal disorders (NDs) are of great importance for evidence-based health care planning in China, whereas such an estimate has been lacking at national level. This study aims to estimate the temporal trends and the attributable burdens of selected risk factors of NDs and their specific causes in China from 1990 to 2019, and to predict the possible trends between 2020 and 2024.

**Methods:**

Data was explored from the Global Burden of Disease study (GBD) 2019. Six measures were used: incidence, mortality, prevalence, disability-adjusted life years (DALYs), years lived with disability (YLDs), and years of life lost (YLLs). Absolute numbers and age-standardized rates (with 95% uncertainty intervals) were calculated. The specific causes of NDs mainly included neonatal preterm birth (NPB), neonatal encephalopathy due to birth asphyxia and trauma (NE), neonatal sepsis and other neonatal infections (NS), and hemolytic disease and other neonatal jaundice (HD). An autoregressive integrated moving average (ARIMA) model was used to forecast disease burden from 2020 to 2024.

**Results:**

There were notable decreasing trends in the number of deaths (84.3%), incidence (30.3%), DALYs (73.5%) and YLLs (84.3%), while increasing trends in the number of prevalence (102.3%) and YLDs (172.7%) from 1990 to 2019, respectively. The corresponding age-standardized rates changed by -74.9%, 0.1%, -65.8%, -74.9%, 86.8% and 155.1%, respectively. Four specific causes of NDs followed some similar and different patterns. The prediction results of the ARIMA model shown that all measures still maintained the original trends in the next five years. Low birth weight, short gestation, ambient particulate matter pollution and household air pollution from solid fuels were the four leading risk factors.

**Conclusion:**

The health burden due to NDs is declining and is likely to continue to decline in the future in China. Delaying the increasing burden of disability may be the next target of concern. Targeted prevention and control strategies for specific causes of NDs are urgently needed to reduce the disease burden.

**Supplementary Information:**

The online version contains supplementary material available at 10.1186/s12889-023-15050-x.

## Introduction

Neonatal health is of significant public health concern worldwide, which reflects multiple aspects of societal wellbeing [1]. Sustainable Development Goal (SDG) 3.2 specifically calls to [2], “By 2030, end preventable deaths of newborn babies and children under five years of age, with all countries aiming to reduce neonatal mortality to at least as low as 12 per 1000 live births and under-five mortality to at least as low as 25 per 1000 live births.” The targets have greatly promoted the development of neonatal health services in most countries and regions around the world [3–5].

Neonatal disorders (NDs) were a critical part of the SDG, and the disease burden of NDs was comprehensively assessed and quantified by the Global Burden of Disease study (GBD). A previous study reported a plateau and decreasing changing trend in the incidence and mortality of NDs globally from 1990 to 2019, respectively, and it indicated that low and middle socio-demographic index (SDI) settings undertook the heaviest burden [6]. The population of China represented almost one fifth of the global population. All the world will be vitally affected by the development of neonatal health in China, even if it affects no one outside China [7]. Besides, the context of rapid ageing and decreasing fertility further complicated the situation of neonatal health care in China [8]. Shao et al. reported for the first time that disease burden indicators for NDs in China showed a decreasing trend between 1990 and 2010, and demonstrated the need to focus on the prognosis of NDs while improving neonatal survival [9]. However, an updated comparison of risk factors and more precise understanding of the disease burden of NDs and their specific causes in China were scarce at national level.

We will address this gap by conducting detailed analysis and reasonable prediction of disease burden through examining incidence, prevalence, death, disability-adjusted life years (DALYs), years lived with disability (YLDs) and years of life lost (YLLs), as well as analyzing corresponding risk factors of NDs at national level. In this study, we have three objectives. First, we aim to present detailed, comprehensive numerical assessment and trend analysis of progress towards the burden of NDs and their specific causes in China over the past three decades to highlight successes and potential focus areas for improvement. Second, we aim to reflect the possible trend of the disease burden of NDs in China in the next five years. Third, we aim to provide vital information and background for prevention and treatment that aim to reduce the disease burden of NDs in China by highlighting the corresponding risk factors.

## Methods

### Overview of GBD 2019 and disease definition

The GBD 2019 provides a systematic and comprehensive assessment for a mutually exclusive and collectively exhaustive list of diseases and injuries at global, national, and subnational levels from 1990 to 2019 [10]. The GBD collaborators collect related data sources from field surveys, censuses, vital statistics, and other health-related data sources. Details of the data, methodology used, and statistical modeling for the GBD 2019 have been described elsewhere [10, 11]. The overall disease burden of NDs and corresponding attributable risk factors from 1990 to 2019 were explored through the Global Health Data Exchange query tool (http://ghdx.healthdata.org/gbd-results-tool). Based on the GBD 2019, the specific causes of NDs mainly included [10]:

1. neonatal preterm birth (NPB);
2. neonatal encephalopathy due to birth asphyxia and trauma (NE);
3. neonatal sepsis and other neonatal infections (NS);
4. hemolytic disease and other neonatal jaundice (HD).

### Measures of disease burden

In this study, we used six measures (including incidence, mortality, prevalence, DALYs, YLDs and YLLs) to reflect the disease burden of NDs. The definition and calculation methods of these measures were described in detail in previous study [10]. We also reported the point estimates and 95% uncertainty interval (UI) in this study. The 95% UI was obtained by repeatedly sampling the sample 1000 times, whose upper and lower bounds were derived based on the 2.5th and 97.5th percentiles of the uncertainty distribution [10]. Besides, age-standardized estimates were performed using a global age structure from 2019 [12].

### Attributable risk factors

Both attributable number and attributable age-standardized rate of death, DALYs, YLDs and YLLs were estimated for the following 5 categories of risk factors: all risk factors, low birth weight, short gestation, ambient particulate matter pollution, and household air pollution from solid fuels. Population attributable fraction (PAF) was calculated by attributable number of selected risk factors based on comparative risk assessment (CRA) [13]. The PAF is defined as the percentage of a disease that will be eliminated if a certain risk factor is eliminated. Attributable burden was calculated by multiplying the cause measure in question by the PAF [13].

### Statistical analysis

The autoregressive integrated moving average (ARIMA) (p, d, q) model was applied to forecast the disease burden of NDs from 2020 to 2024. Three parameters (p, d, and q) represented the orders of autoregression, degree of difference, and order of moving average, respectively. More details of this model were described elsewhere [14]. We first performed logarithmic transformation on the original data and differential processing on the time series (if necessary). The Augmented Dickey–Fuller (ADF) test was used to ensure that the series was stationary, and the autocorrelation function (ACF) and the partial autocorrelation function (PACF) were used to identify the appropriate model parameters (p and q). We selected the best ARIMA (p, d, q) models by the Akaike information criterion (AIC) and the Bayesian information criterion (BIC) to predict the disease burden of NDs from 2020 to 2024. The Ljung–Box Q test was used to determine that the residuals of the selected models satisfied an independent normal distribution.

All the analyses and graphics were produced with the R statistical program (version 4.0.3).

## Results

### Mortality, incidence and prevalence

**Fig. 1:**
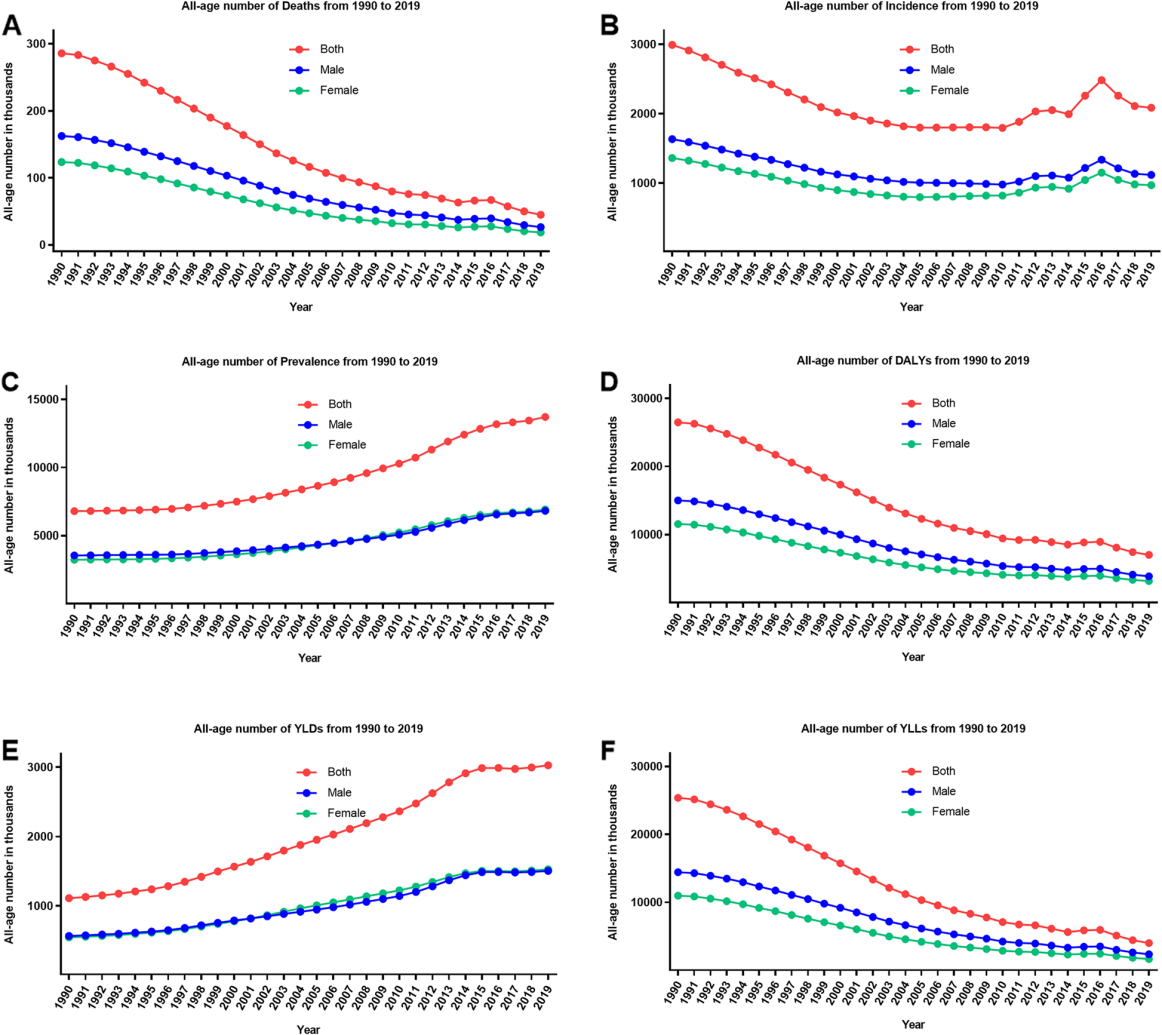
The trend of all-age number of all six measures for neonatal disorders in China from 1990 to 2019. **A** Deaths; **B** incidence; **C** prevalence; **D** DALYs; **E** YLDs; **F** YLLs

**Fig. 2:**
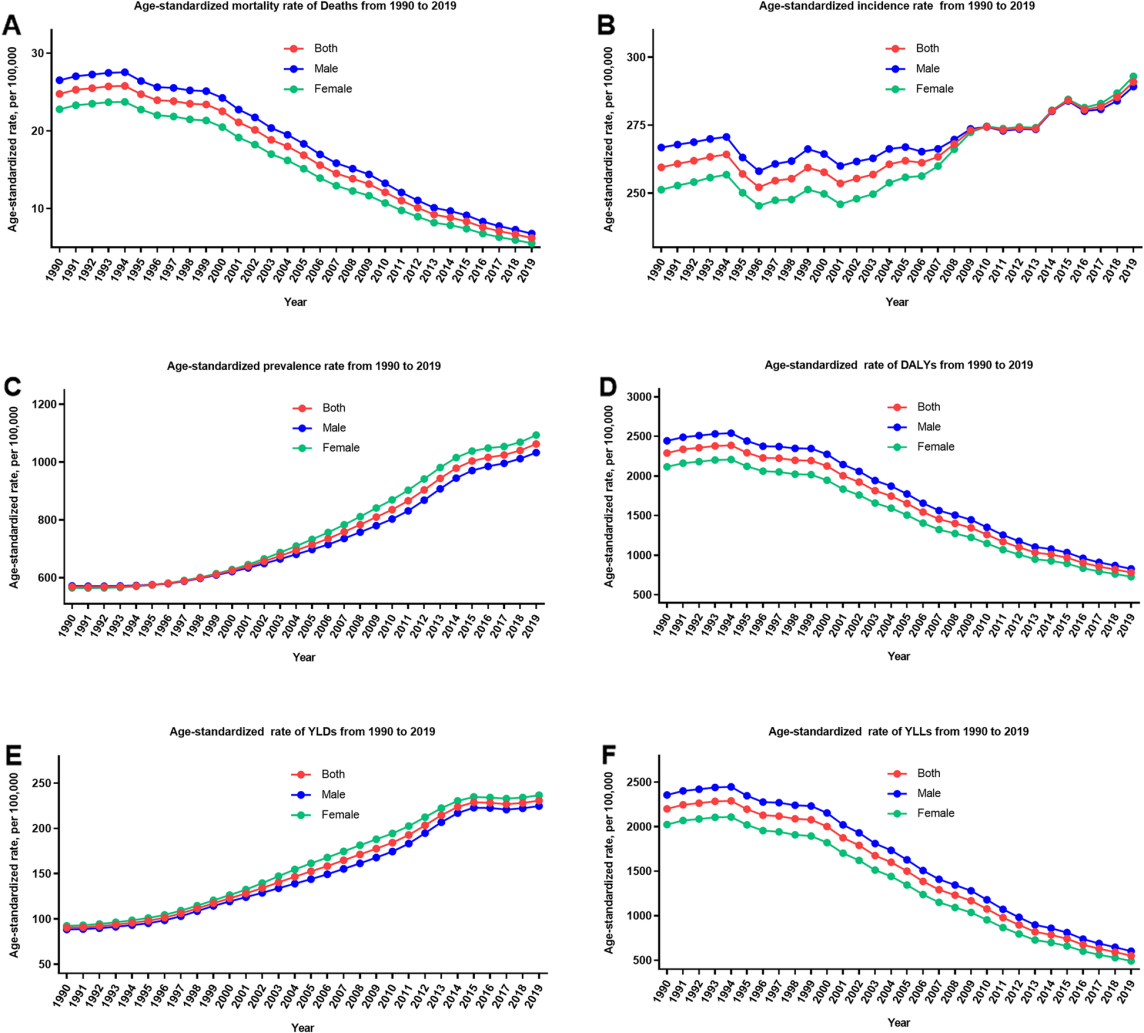
The trend of age-standardized rate of all six measures for neonatal disorders in China from 1990 to 2019. **A** Mortality rate; **B** incidence rate; **C** prevalence rate; **D** DALYs rate; **E** YLDs rate; **F** YLLs rate

Table 1; Figs. 1 (A, B, C) and 2 (A, B, C) showed the overall number and age-standardized rate of NDs and percentage changes by gender in China from 1990 to 2019. Over the past three decades studied here, mortality and incidence decreased: mortality by 84.3% (95% UI: 81.1–87.0%), incidence by 30.3% (24.4–35.9%); while prevalence increased by 102.3% (78.6–132.8%). In 1990, the estimated numbers of deaths, incidence, and prevalence nationwide in China from NDs were 0.29 million (0.25 to 0.32), 2.99 million (2.61 to 3.44), and 6.78 (6.16 to 7.38), respectively. In 2019, the values of the above three measures were 0.04 million (0.04 to 0.05), 2.09 (1.72 to 2.52), and 13.72 (11.98 to 15.74). As for different gender groups, the number of deaths for males were higher than for females. However, there are no significant differences in the number of incidence and prevalence between males and females. Similar patterns can be observed in the four specific causes of NDs (Tables S1-S4, Figs. S1-S4 (A, B, C)).

**Table 1:** All-age number and age-standardized rate of all measures for neonatal disorders and percentage changes by gender in China, 1990 and 2019

Measures	All-age number in thousands (95% UI)			Age-standardized rate, per 100,000 (95% UI)		
	1990	2019	Change, %	1990	2019	Change, %
Deaths						
Total	285.9 (254.2, 319.8)	44.9 (38.4, 52.0)	-84.3 (-87.0, -81.1)	24.8 (22.0, 27.7)	6.2 (5.3, 7.2)	-74.9 (-79.3, -69.8)
Male	162.4 (142.6, 183.1)	26.5 (22.1, 31.0)	-83.7 (-87.0, -80.1)	26.5 (23.3, 29.9)	6.8 (5.7, 7.9)	-74.4 (-79.5, -68.6)
Female	123.5 (110.6, 137.0)	18.5 (21.0, 16.0)	-85.0 (-87.4, -82.2)	22.8 (20.4, 25.3)	5.6 (4.8, 6.3)	-75.6 (-79.5, -71.0)
Incidence						
Total	2991.5 (2617.0, 3441.2)	2085.6 (1721.2, 2517.7)	-30.3 (-35.9, -24.4)	259.5 (227.0, 298.5)	290.9 (240.2, 350.8)	0.1 (-3.1, 21.5)
Male	1631.6 (1428.8, 1886.0)	1117.1 (922.3, 1348.1)	-31.5 (-37.0, -25.9)	266.8 (233.6, 308.3)	289.2 (238.9, 348.6)	8.4 (-0.3, -17.2)
Female	1359.9 (1194.7, 1557.4)	968.5 (800.1, 1173.1)	-28.8 (-35.0, -22.5)	251.2 (220.7, 287.7)	292.9 (242.0, 354.5)	16.6 (6.3, 26.9)
Prevalence						
Total	6784.4 (6155.3, 7382.4)	13717.0 (11982.3, 15739.2)	102.3 (78.6, 132.8)	568.7 (516.8, 618.4)	1062.5 (936.0, 1213.5)	86.8 (65.5, 113.8)
Male	3544.5 (3203.8, 3900.2)	6803.9 (5976.5, 7846.8)	92.0 (68.1, 122.8)	571.9 (517.7, 629.1)	1032.9 (911.4, 1185.6)	80.6 (58.9, 108.4)
Female	3236.9 (2909.9, 3560.4)	6913.1 (6027.6, 7991.0)	113.6 (85.6, 147.8)	565.3 (509.0, 621.4)	1093.3 (961.4, 1259.0)	93.4 (69.1, 122.7)
DALYs						
Total	26489.9 (23582.5, 29517.7)	7017.5 (6148.7, 8043.7)	-73.5 (-77.5, -68.8)	2289.8 (2036.3, 2552.2)	782.6 (698.6, 886.7)	-65.8 (-70.7, -59.7)
Male	14980.6 (13209.1, 16833.8)	3850.2 (3368.5, 4408.6)	-74.3 (-78.5, -69.3)	2444.0 (2155.2, 2747.5)	827.4 (718.9, 947.8)	-66.1 (-71.8, -59.4)
Female	11509.3 (10340.2, 12724.2)	3618.6 (2770.2, 3618.6)	-72.5 (-76.7, -67.8)	2115.4 (1901.9, 2340.0)	729.3 (650.1, 819.6)	-65.5 (-70.3, -60.0)
YLDs						
Total	1110.6 (844.5, 1454.6)	3028.3 (2339.2, 3829.3)	172.7 (121.6, 234.4)	90.4 (68.7, 117.9)	230.6 (177.0, 291.7)	155.1 (108.5, 211.3)
Male	565.9 (427.4, 746.9)	1502.6 (1154.4, 1893.7)	165.5 (115.6, 227.3)	88.6 (67.0, 116.5)	224.6 (173.2, 283.5)	153.6 (105.8, 211.2)
Female	544.7 (416.2, 711.2)	1525.7 (1175.6, 1938.0)	180.1 (128.0, 240.1)	92.4 (70.7, 120.6)	236.6 (182.3, 298.9)	156.2 (109.9, 211.3)
YLLs						
Total	25379.3 (22564.9, 28384.9)	3989.2 (3409.9, 4610.7)	-84.3 (-87.0, -81.1)	2199.4 (1955.4, 2459.7)	552.0 (471.8, 637.9)	-74.9 (-79.3, -69.8)
Male	14414.7 (12664.0, 16254.0)	2347.6 (1957.3, 2748.7)	-83.7 (-87.0, -80.1)	2355.4 (2069.5, 2655.7)	602.7 (502.7, 705.5)	-74.4 (-79.5, -68.7)
Female	10964.6 (9821.5, 12161.0)	1641.6 (1424.6, 1865.0)	-85.0 (-87.4, -82.2)	2023.0 (1812.4, 2244.2)	492.7 (427.8, 559.6)	-75.6 (-79.5, -71.0)

*Abbreviations: UI* Uncertainty interval, *DALYs* Disability-adjusted life years, *YLDs* Years lived with disability, *YLLs* Years of life lost

When standardized by age, mortality, incidence and prevalence rate changed differently, there were both increases and decreases. The age-standardized mortality rate decreased by 74.9% (69.8–79.3%) during these 30 years, while the age-standardized incidence and prevalence rate increased by 0.1% (-3.1–21.5%) and 86.8% (65.5–113.8%) during these 30 years, respectively. In addition, it can be observed that the age-standardized incidence rate of NDs showed an unstable fluctuation over the past three decades. NS, one specific cause of NDs, showed an obvious growth trend, whereas other subcategories of NDs showed downward trends. Some different patterns can also be seen in the subcategories of NDs (Tables S1-S4, Figs. S5-S8 (A, B, C)).

### DALYs, YLDs and YLLs

Over the past three decades, the number of all-age DALYs and YLLs of NDs decreased by 73.5% (68.8–77.5%), and 84.3% (81.1–87.0%), respectively, while the number of all-age YLDs increased by 172.7% (121.6–234.4%) (Table 1; Figs. 1 (D, E, F) and 2 (D, E, F)). The number of DALYs decreased from 26.49 million (23.58 to 29.52) in 1990 to 7.02 million (6.15 to 8.04) in 2019. The age-standardized DALYs rates decreased by 65.8% (59.7–70.7%) from 1990 to 2019. Likewise, the number of YLLs decreased from 25.38 million (22.56 to 28.38) in 1990 to 3.99 million (3.41 to 4.61) in 2019. The age-standardized YLLs rates decreased by 74.9% (69.8–79.3%) from 1990 to 2019. However, the raw number of YLDs followed a different pattern, increasing from 1.11 million (0·84 to 1.45) in 1990 to 3.03 million (2.34 to 3.83) in 2019. The age-standardized YLDs rate increased by 155.1% (108.5–211.3%) from 1990 to 2019. Over the past three decades, the number of DALYs and YLLs for males were higher than for females. However, once counts were converted to age-standardized rates, these differences of all measures were not observed. Further, four specific causes of NDs followed some similar and different patterns (Tables S1-S4, Figs. S1-S8 (D, E, F)).

### Prediction of age-standardized rate by ARIMA models

In this study, the data and time series needed to be transformed and differentiated to make them steady-state sequences, which supported by the results of the ADF test. The selected optimal ARIMA model parameters and corresponding AIC and BIC were described in the Table S9. The residuals of all models failed to pass the significance test, which indicated that the selected models can well fit the data. The forecast results of age-standardized rate from 2020 to 2024 were shown in Table 2. From 2020 to 2024, the age-standardized rate of mortality, DALYs and YLLs of NDs showed downward trends, and their estimated values were predicted to decrease to 3.9 per 100,000 (-1.7 to 9.6), 592.4 per 100,000 (83.5 to 1101.2), and 349.0 per 100,000 (-156.1 to 854.1) in 2024, respectively. On the contrary, the age-standardized rate of incidence, prevalence and YLDs would increase to 300.1 per 100,000 (275.8 to 324.5), 1227.7 per 100,000 (1057.5 to 1397.8) and 245.7 per 100,000 (224.0 to 267.4) in 2024, respectively. The forecast results for four specific causes of NDs from 2020 to 2024 were shown in Tables S5-S8.

**Table 2:** Prediction of age-standardized rate (per 100,000) of all six measures for neonatal disorders for the next 5 years according to ARIMA models with 95% confidence interval in China

Measures	2020	2021	2022	2023	2024
Deaths					
Both	5.7 (4.9, 6.5)	5.3 (3.5, 7.0)	4.8 (1.9, 7.7)	4.3 (0.1, 8.5)	3.9 (-1.7, 9.6)
Male	6.2 (5.4, 7.1)	5.7 (3.9, 7.6)	5.2 (2.2, 8.3)	4.7 (0.2, 9.2)	4.2 (-1.8, 10.3)
Female	5.1 (4.4, 5.8)	4.7 (3.1, 6.3)	4.3 (1.6, 6.9)	3.9 (0, 7.7)	3.5 (-1.6, 8.7)
Incidence					
Both	295.1 (289.3, 301.0)	296.4 (283.7, 309.1)	297.6 (280.4, 314.8)	298.9 (277.9, 319.9)	300.1 (275.8, 324.5)
Male	290.6 (284.2, 297.1)	292.1 (282.6, 301.7)	293.6 (281.3, 306.0)	295.1 (280.3, 310.0)	296.6 (279.3, 314.0)
Female	297.7 (291.8, 303.6)	299.7 (286.9, 312.5)	301.7 (284.1, 319.3)	303.7 (282.0, 325.3)	305.6 (280.2, 331.1)
Prevalence					
Both	1088.5 (1082.0, 1094.9)	1118.0 (1094.0, 1142.0)	1151.0 (1095.4, 1206.6)	1187.6 (1084.0, 1291.2)	1227.7 (1057.5, 1397.8)
Male	1057.1 (1050.5, 1063.8)	1084.9 (1061.2, 1108.5)	1116.0 (1062.4, 1169.5)	1150.5 (1051.9, 1249.0)	1188.4 (1027.8, 1348.9)
Female	1123.0 (1115.9, 1130.1)	1158.3 (1132.0, 1184.6)	1199.2 (1138.5, 1259.8)	1245.6 (1132.8, 1358.4)	1297.7 (1112.5, 1482.8)
DALYs					
Both	744.5 (675.9, 813.1)	706.5 (553.0, 859.9)	668.4 (411.7, 925.2)	630.4 (254.6, 1006.2)	592.4 (83.5, 1101.2)
Male	785.4 (711.8, 859.1)	743.5 (578.9, 908.2)	701.6 (426.1, 977.1)	659.7 (256.5, 1063.0)	617.8 (71.8, 1163.9)
Female	695.7 (632.9, 758.4)	662.1 (521.7, 802.5)	628.5 (393.6, 863.4)	595.0 (251.1, 938.8)	561.4 (95.8, 1027.0)
YLDs					
Both	232.7 (230.9, 234.5)	235.2 (229.1, 241.3)	238.3 (226.8, 249.9)	241.9 (225.1, 258.7)	245.7 (224.0, 267.4)
Male	226.3 (224.3, 228.3)	228.1 (221.6, 234.7)	230.8 (218.5, 243.1)	233.9 (216.1, 251.8)	237.4 (214.3, 260.4)
Female	239.9 (238.1, 241.8)	244.0 (238.3, 249.6)	248.5 (238.0, 259.0)	253.4 (237.7, 269.0)	258.3 (237.9, 278.7)
YLLs					
Both	511.3 (443.2, 579.4)	470.7 (318.4, 623.0)	430.1 (175.3, 685.0)	389.6 (16.5, 762.6)	349.0 (-156.1, 854.1)
Male	558.3 (485.3, 631.3)	513.9 (350.7, 677.2)	469.5 (196.4, 742.7)	425.2 (25.3, 825.0)	380.8 (-160.5, 922.2)
Female	456.4 (394.0, 518.9)	420.3 (280.7, 559.9)	384.1 (150.6, 617.7)	348.0 (6.1, 689.8)	311.8 (-151.0, 774.7)

*Abbreviations: DALYs* Disability-adjusted life years, *YLDs* Years lived with disability, *YLLs* Years of life lost

### Attributable burden by selected risk factors

**Fig. 3:**
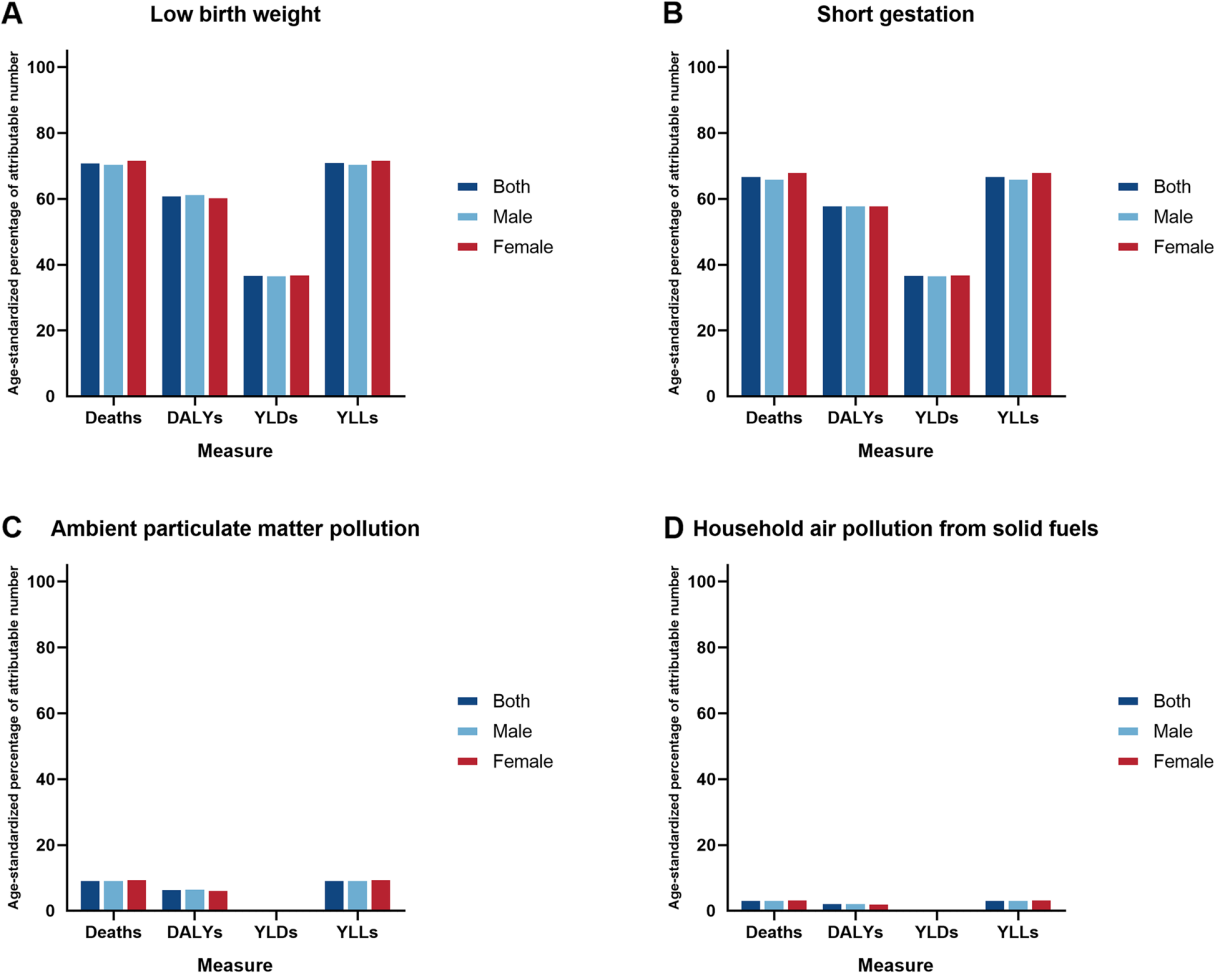
Age-standardized percentage of attributable number of risk factors of four measures for neonatal disorders by gender in China, 2019. **A** low birth weight; **B** short gestation; **C** ambient particulate matter pollution; **D** household air pollution from solid fuels

Attributable number and attributable age-standardized rate of mortalities, DALYs, YLDs and YLLs were estimated in different gender groups in 2019 for the 5 risk factors examined here as shown in Table 3. The attributable number of deaths for NDs including all risk factors was 0.04 million (0.03 to 0.04). For DALYs, it was 4.26 million (3.79 to 4.84). For YLDs, it was 1.12 million (0.87 to 1.40), and 3.13 million (2.68 to 3.63) for YLLs. The attributable age-standardized rates of mortalities, DALYs, YLDs and YLLs were 4.9 (4.1 to 5.6), 520.6 (458.9 to 594.0), 85.3 (66.5 to 106.4), and 435.2 (372.3 to 504.7) per 100,000 in 2019, respectively. Of these four subtypes of all risk factors, low birth weight and short gestation were the two major risk factors for NDs of all the four indicators in both attributable number or attributable age-standardized rate (Table 3; Fig. 3). These results were regardless of gender. This pattern was consistent in four specific causes of NDs (Tables S10-S13).

**Table 3:** Points estimated and 95% uncertainty interval of attributable number and age-standardized rate of risk factors for neonatal disorders by gender in China, 2019

Measures	All risk factors	Low birth weight	Short gestation	Ambient particulate matter pollution	Household air pollution from solid fuels
**Attributable number in thousands**					
Deaths					
Both	35.3 (30.2, 40.9)	32.0 (27.3, 37.1)	30.1 (25.8, 35.0)	4.4 (3.3, 5.5)	1.7 (1.0, 2.6)
Female	14.6 (12.7, 16.5)	13.3 (11.6, 15.2)	12.6 (11.0, 14.4)	1.8 (1.3, 2.4)	0.7 (0.4, 1.1)
Male	20.7 (17.3, 24.5)	18.7 (15.7, 22.1)	17.5 (14.8, 20.8)	2.5 (1.8, 3.4)	1.0 (0.5, 1.6)
DALYs					
Both	4256.8 (3791.8, 4840.8)	3966.2 (3526.5, 4504.3)	3799.8 (3376.5, 4328.6)	387.3 (292.4, 487.6)	148.6 (84.8, 235.1)
Female	1865.7 (1663.2, 2098.6)	1752.7 (1553.4, 1977.7)	1693.0 (1496.6, 1910.3)	161.9 (116.9, 209.2)	61.6 (33.9, 99.0)
Male	2391.0 (2092.4, 2747.7)	2213.5 (1941.6, 2537.3)	2106.7 (1843.2, 2423.0)	225.4 (160.8, 298.4)	87.0 (47.0, 140.0)
YLDs					
Both	1123.5 (873.9, 1404.5)	1123.1 (873.4, 1404.0)	1123.1 (873.4, 1404.0)	0.6 (0.4, 0.8)	0.2 (0.1, 0.3)
Female	571.1 (444.7, 718.2)	570.8 (444.5, 718.0)	570.8 (444.5, 718.0)	0.3 (0.2, 0.4)	0.1 (0.1, 0.2)
Male	552.3 (431.5, 692.1)	552.3 (431.5, 692.1)	552.3 (431.5, 692.1)	0.3 (0.2, 0.5)	0.1 (0.1, 0.2)
YLLs					
Both	3133.2 (2680.6, 3632.0)	2843.1 (2424.9, 3289.6)	2676.7 (2288.1, 3108.8)	386.7 (291.6, 486.8)	148.4 (84.7, 234.8)
Female	1294.7 (1126.6, 1469.7)	1181.8 (1030.1, 1345.6)	1122.2 (973.4, 1282.6)	161.6 (116.6, 208.9)	61.6 (33.9, 98.9)
Male	1838.5 (1534.1, 2171.2)	1661.2 (1391.4, 1962.4)	1554.5 (1294.6, 1844.4)	225.1 (160.4, 298.1)	86.8 (46.9, 139.8)
**Attributable age-standardized rate per 100,000**					
Deaths					
Both	4.9 (4.1, 5.6)	4.4 (3.7, 5.1)	4.1 (3.5, 4.8)	0.6 (0.4, 0.7)	0.2 (0.1, 0.3)
Female	4.3 (3.8, 4.9)	4.0 (3.4, 4.5)	3.8 (3.2, 4.3)	0.5 (0.3, 0.7)	0.2 (0.1, 0.3)
Male	5.3 (4.4, 6.3)	4.8 (4.0, 5.6)	4.5 (3.7, 5.3)	0.6 (0.4, 0.8)	0.3 (0.1, 0.4)
DALYs					
Both	520.6 (458.9, 594.0)	480.0 (424.3, 547.2)	456.9 (403.2, 521.7)	54.0 (40.7, 68.0)	20.7 (11.8, 32.7)
Female	477.9 (425.9, 536.9)	443.7 (397.9, 500.5)	425.8 (381.0, 479.9)	48.9 (35.3, 63.2)	18.6 (10.2, 29.9)
Male	556.7 (480.7, 644.9)	510.8 (441.0, 589.6)	483.1 (417.4, 561.5)	58.3 (41.6, 77.2)	22.5 (12.1, 36.2)
YLDs					
Both	85.3 (66.5, 106.4)	85.2 (66.4, 106.3)	85.2 (66.4, 106.3)	0.1 (0.1, 0.1)	0 (0, 0)
Female	87.9 (68.0, 109.9)	87.8 (68.0, 109.8)	87.8 (68.0, 109.8)	0.1 (0.1, 0.1)	0 (0, 0)
Male	82.7 (64.2, 103.3)	82.7 (64.2, 103.2)	82.7 (64.2, 103.2)	0.1 (0.1, 0.1)	0 (0, 0)
YLLs					
Both	435.2 (372.3, 504.7)	394.8 (336.7, 456.3)	371.6 (317.7, 431.5)	53.9 (40.7, 67.9)	20.6 (11.8, 32.7)
Female	390.0 (339.4, 442.9)	355.9 (310.3, 405.3)	337.9 (293.0, 386.2)	48.8 (35.3, 63.2)	18.6 (10.2, 29.8)
Male	473.9 (395.6, 559.6)	428.0 (358.5, 505.5)	400.4 (333.4, 475.0)	58.3 (41.5, 77.2)	22.4 (12.1, 36.1)

*Abbreviations: DALYs* Disability-adjusted life years, *YLDs* Years lived with disability, *YLLs* Years of life lost

## Discussion

This population-based nationwide epidemiologic study provided an updated information for disease burden of NDs in China from 1990 to 2019. We found that the burden of death from NDs decreased substantially, while the burden of disability increased from year 1990 to 2019. In addition, the present research predicted that this trend will be continued in the next five years.

NDs may have the same impact on males and females in China. Mounting evidence seemed to provide some conflicting information about gender difference in neonatal mortality [15–17]. However, a study from South Asia highlighted an important point of stratified analysis by early and late neonatal period [18]. The gender differences in the burden of NDs may be affected by physiological and genetic factors, as well as social factors [18]. Considering the results of our current study, it is not clear whether there is a similar gender difference pattern of NDs in China.

A previous study reported that the incident trend of NDs was relatively stable (changes: -0.26%), while the burden of death showed a downward trend (changes: 37.38%) globally from 1990 to 2019 [6]. China has also made encouraging progress in reducing the burden of NDs over the past three decades in this study. Neonatal health is an essential part of assessing the health status of a country or region as it is the starting point of life, which is closely linked with the health of the whole lifecycle. This focus is integrated in the visions of both the SDG 3.2 and the Healthy China 2030 agenda [8]. Of 204 countries, a reference scenario suggested that, by 2030, 139 (68%) were likely to meet the neonatal mortality rate SDG target [1]. In fact, more than 90% of countries have the potential to achieve the SDG target by strengthening quality health systems, scaling up interventions, addressing within-country disparities, and pursuing integrative action on social determinants of health [1, 5]. China provides an important reference for neonatal health in many developing countries around the world, and the most notable contributors in China to the achievements in neonatal health are strong political will to focus on newborns and improvements in gender equity [19]. Another major achievement is the establishment of national information systems of maternal and child health, which provide information for the development of key maternal and child health policies in China, including social health insurance and poverty alleviation [8, 20]. Increased hospital delivery rates, improved resuscitation skills, and the development of neonatal intensive care have all contributed to the reduction of neonatal deaths [8], but the burden of disease in the neonatal stage requires not only improving neonatal survival but also requires further attention. It is worth noting that delaying the increasing burden of disability may be China’s next priority.

In this study, the specific causes of NDs were also explored. Inconsistent patterns of disease burden were observed in some subcategories of NDs, suggesting differences in etiology and pathophysiology among these causes. Therefore, the current study highlights the importance of targeted measures for the control and management of specific causes of NDs. NPB is a major contributor to the heavy disease burden of NDs. The low preterm birth rate in pregnant women might have benefited from health care reform over the past decade in China [12]. However, the universal two-child policy came into effect on October 29, 2015, which may have contributed to the increase in the number of NPB (Figure S1 (B)). Many cases of NE, and the often-resultant neonatal death or stillbirth, are likely to result from a complex multifactorial pathway to brain injury to which hypoxia-ischemia substantially contributes [21]. The universal two-child policy also further complicated the epidemiological characteristics of NS in China. It can be observed that the main contributor of disease burden of NS changed from death to disability over the past three decades (Figure S3 (E, F)). Ameliorating the possible complications of NS may be a major goal in reducing the burden of disease [22]. Evidently, although HD may be less prevalent than entities like other specific causes, it remains an important way to reduce the burden of NDs.

The present study suggests that paying more attention to low birth weight, short gestation is a key way to reduce the burden of NDs. In preterm babies with very low birth weight, perinatal hypoxia induced by immature lung development, leads to abnormal growth and maturation of susceptible cell types, particularly neurons and oligodendrocytes, which are associated with decreased cerebral and cerebellar volumes and increases in cerebral ventricular size [23]. In preterm and very low birth weight infants, the important pathogens associated with early-onset NS are Streptococcus agalactiae and Escherichia coli [22]. Besides, oxidative stress, DNA methylation, mitochondrial DNA content alteration, and endocrine disruptions may all play an important role in environmental pollution induced adverse effects to pregnant women and fetuses [24]. Limited by incomplete survey data and lack of various types of indicators, additional measures for specific causes should also be considered. Basic activities include chlorhexidine umbilical cord cleansing for NS prevention, intensive care for prematurity, and advanced resuscitation for intrapartum asphyxia [1, 25, 26]. A recent study from China showed a high level of Particulate Matter 2.5 (PM 2.5) pollution exposure in China, and environmental PM 2.5 concentration was positively and strongly correlated with neonatal disease burden [27]. Even though the burden of NDs attributable to ambient particulate matter pollution had declined significantly, China still had a serious environmental pollution problem. Several studies had also shown that PM 2.5 exposure during pregnancy had adverse effects on neonatal health and resulted in adverse outcomes such as preterm birth, low birth weight, neonatal death, and impaired lung development [28–30]. Further increases in environmental pollution treatment and control may be more helpful in reducing the burden of NDs.

This study provided timely information on the incidence, prevalence, mortality, DALYs, YLDs, YLLs and the main risk factors of NDs in China from 1990 to 2019, and reasonably predicted the disease burden in the next five years. The methodology was widely used in GBD studies and had shown its robustness. Admittedly, several limitations should be acknowledged. First, the limitations of the GBD 2019 still exist, which have been described in mounting previous study. Second, specific reasons for regional and provincial disease burden differences remain unclear and require more further investigation. There are still differences in health issues and access to health care providers and services among provinces, and evidence-based health decision-making at the provincial level is crucial in China. Third, our future health scenario analyses are benchmarked against past trends, suggesting that the predicted values are limited in its ability to capture disruptions that could arise as a consequence of future crises, such as the COVID-19 pandemic. Thankfully, although children have been found to be at risk of developing multisystem inflammatory syndrome as result of COVID-19 pandemic, they appear to be less at risk of severe illness and death [1, 31]. However, there is still a need to respond to the negative impact of the COVID-19 pandemic on newborns.

## Conclusion

The health burden due to NDs is declining and is likely to continue to decline in the future in China. Delaying the increasing burden of disability may be the next target of concern. More attention should be paid to the management of risk factors including low birth weight, short gestation, ambient particulate matter pollution and household air pollution from solid fuels. Targeted prevention and control strategies for specific causes of NDs are urgently needed to reduce the disease burden.

## Supplementary Information

**Additional file 1: Table S1.** All-age number and age-standardized rate of all measures for neonatal preterm birth and percentage changes by gender in China, 1990 and 2019. **Table S2.** All-age number and age-standardized rate of all measures for neonatal encephalopathy due to birth asphyxia and trauma and percentage changes by gender in China, 1990 and 2019. **Table S3.** All-age number and age-standardized rate of all measures for neonatal sepsis and other neonatal infections and percentage changes by gender in China, 1990 and 2019. **Table S4.** All-age number and age-standardized rate of all measures for hemolytic disease and other neonatal jaundice and percentage changes by gender in China, 1990 and 2019. **Table S5.** Prediction of age-standardized rate (per 100,000) of all six measures for neonatal preterm birth for the next 5 years according to ARIMA models with 95% confidence interval in China. **Table S6.** Prediction of age-standardized rate (per 100,000) of all six measures for neonatal encephalopathy due to birth asphyxia and trauma for the next 5 years according to ARIMA models with 95% confidence interval in China. **Table S7.** Prediction of age-standardized rate (per 100,000) of all six measures for neonatal sepsis and other neonatal infections for the next 5 years according to ARIMA models with 95% confidence interval in China. **Table S8.** Prediction of age-standardized rate (per 100,000) of all six measures for hemolytic disease and other neonatal jaundice for the next 5 years according to ARIMA models with 95% confidence interval in China. **Table S9.** ARIMA model parameters and their corresponding AIC and BIC for prediction of age-standardized rate (per 100,000) of all six measures for neonatal disorders for the next 5 years in China. **Table S10.** Points estimated and 95% uncertainty interval of attributable number and age-standardized rate of risk factors for neonatal preterm birth by gender in China, 2019. **Table S11.** Points estimated and 95% uncertainty interval of attributable number and age-standardized rate of risk factors for neonatal encephalopathy due to birth asphyxia and trauma by gender in China, 2019. **Table S12.** Points estimated and 95% uncertainty interval of attributable number and age-standardized rate of risk factors for neonatal sepsis and other neonatal infections by gender in China, 2019. **Table S13.** Points estimated and 95% uncertainty interval of attributable number and age-standardized rate of risk factors for hemolytic disease and other neonatal jaundice by gender in China, 2019. **Figure S1.** The trend of all-age number of all six measures for neonatal preterm birth in China from 1990 to 2019. **Figure S2.** The trend of all-age number of all six measures for neonatal encephalopathy due to birth asphyxia and trauma in China from 1990 to 2019. **Figure S3.** The trend of all-age number of all six measures for neonatal sepsis and other neonatal infections in China from 1990 to 2019. **Figure S4.** The trend of all-age number of all six measures for hemolytic disease and other neonatal jaundice in China from 1990 to 2019. **Figure S5.** The trend of age-standardized rate of all six measures for neonatal preterm birth in China from 1990 to 2019. **Figure S6.** The trend of age-standardized rate of all six measures for neonatal encephalopathy due to birth asphyxia and trauma in China from 1990 to 2019. **Figure S7.** The trend of age-standardized rate of all six measures for neonatal sepsis and other neonatal infections in China from 1990 to 2019. **Figure S8.** The trend of age-standardized rate of all six measures for hemolytic disease and other neonatal jaundice in China from 1990 to 2019. **Figure S9.** Age-standardized percentage of attributable number of risk factors of four measures for neonatal preterm birth by gender in China, 2019. **Figure S10.** Age-standardized percentage of attributable number of risk factors of four measures for neonatal encephalopathy due to birth asphyxia and trauma by gender in China, 2019. **Figure S11.** Age-standardized percentage of attributable number of risk factors of four measures for neonatal sepsis and other neonatal infections by gender in China, 2019. **Figure S12.** Age-standardized percentage of attributable number of risk factors of four measures for hemolytic disease and other neonatal jaundice by gender in China, 2019.

**Additional file 2.**

## Data Availability

The datasets generated during and/or analyzed during the current study are available in the GBD Data Tool repository (http://ghdx.healthdata.org/gbd-results-tool). This public link to the database of GBD study is open, and the use of data does not require additional consent from IHME.

## Abbreviations

SDG: Sustainable Development Goal
NDs: Neonatal disorders
GBD: The Global Burden of Disease study
SDI: Socio-demographic index
DALYs: Disability-adjusted life years
YLDs: Years lived with disability
YLLs: Years of life lost
NPB: Neonatal preterm birth
NE: Neonatal encephalopathy due to birth asphyxia and trauma
NS: Neonatal sepsis and other neonatal infections
HD: Hemolytic disease and other neonatal jaundice
UI: Uncertainty interval
PAF: Population attributable fraction
CRA: Comparative risk assessment
ARIMA: Autoregressive integrated moving average
ADF: Augmented Dickey–Fuller
ACF: Autocorrelation function
PACF: Partial autocorrelation function
AIC: Akaike information criterion
BIC: Bayesian information criterion
PM 2.5: Particulate matter 2.5

## References

[1] Collaborators GU-M (2021). Global, regional, and national progress towards sustainable development goal 3.2 for neonatal and child health: all-cause and cause-specific mortality findings from the global burden of Disease Study 2019. Lancet.

[2] Collaborators GS (2018). Measuring progress from 1990 to 2017 and projecting attainment to 2030 of the health-related Sustainable Development Goals for 195 countries and territories: a systematic analysis for the global burden of Disease Study 2017. Lancet.

[3] Wang H (2014). Global, regional, and national levels of neonatal, infant, and under-5 mortality during 1990–2013: a systematic analysis for the global burden of Disease Study 2013. Lancet.

[4] Collaborators GCoD (2018). Global, regional, and national age-sex-specific mortality for 282 causes of death in 195 countries and territories, 1980–2017: a systematic analysis for the global burden of Disease Study 2017. Lancet.

[5] Collaborators GCM (2016). Global, regional, national, and selected subnational levels of stillbirths, neonatal, infant, and under-5 mortality, 1980–2015: a systematic analysis for the global burden of Disease Study 2015. Lancet.

[6] Ou Z (2022). Global trends in incidence and death of neonatal disorders and its specific causes in 204 countries/territories during 1990–2019. BMC Public Health.

[7] Hesketh T, Wei XZ (1997). Health in China. From Mao to market reform. BMJ.

[8] Qiao J (2021). A Lancet Commission on 70 years of women’s reproductive, maternal, newborn, child, and adolescent health in China. Lancet.

[9] Shao H, et al. Burden of neonatal diseases in China,1990–2010. Dis Surveill. 2015;30(08):663-667.

[10] Collaborators GDaI (2020). Global burden of 369 diseases and injuries in 204 countries and territories, 1990–2019: a systematic analysis for the global burden of Disease Study 2019. Lancet.

[11] Collaborators GRF (2020). Global burden of 87 risk factors in 204 countries and territories, 1990–2019: a systematic analysis for the global burden of Disease Study 2019. Lancet.

[12] Deng K (2021). Preterm births in China between 2012 and 2018: an observational study of more than 9 million women. Lancet Glob Health.

[13] Murray CJ (2003). Comparative quantification of health risks conceptual framework and methodological issues. Popul Health Metr.

[14] Wei J, et al. Time trends in the incidence of spinal Pain in China, 1990 to 2019 and its prediction to 2030: the Global Burden of Disease Study 2019. Pain Ther. 2022;11(4):1245-1266. 10.1007/s40122-022-00422-9 PMC9633916 35969366

[15] Steen EE (2014). Impact of sex on perinatal mortality and morbidity in twins. J Perinat Med.

[16] Speakman JR (2013). Sex- and age-related mortality profiles during famine: testing the ‘body fat’ hypothesis. J Biosoc Sci.

[17] Mishra SK (2018). BIRTH ORDER, STAGE OF INFANCY AND INFANT MORTALITY IN INDIA. J Biosoc Sci.

[18] Aghai ZH (2020). Gender variations in neonatal and early infant mortality in India and Pakistan: a secondary analysis from the Global Network maternal Newborn Health Registry. Reprod Health.

[19] Li L, Fu H (2017). China’s health care system reform: Progress and prospects. Int J Health Plann Manage.

[20] Barber SL, Yao L (2011). Development and status of health insurance systems in China. Int J Health Plann Manage.

[21] Tann CJ (2017). Neonatal Encephalopathy with Group B Streptococcal Disease Worldwide: systematic review, Investigator Group Datasets, and Meta-analysis. Clin Infect Dis.

[22] Shane AL, Sánchez PJ, Stoll BJ (2017). Neonatal sepsis Lancet.

[23] Salmaso N (2014). Neurobiology of premature brain injury. Nat Neurosci.

[24] Li Z (2019). Impact of ambient PM(2.5) on adverse birth outcome and potential molecular mechanism. Ecotoxicol Environ Saf.

[25] Bhutta ZA (2014). Can available interventions end preventable deaths in mothers, newborn babies, and stillbirths, and at what cost?. Lancet.

[26] Akseer N (2015). Ending preventable newborn deaths in a generation. Int J Gynaecol Obstet.

[27] Yuan J, et al. The burden of neonatal diseases attributable to Ambient PM 2.5 in China from 1990 to 2019. Front Environ Sci. 2022;10:828408.

[28] Macchi C (2021). Maternal exposure to air pollutants, PCSK9 levels, fetal growth and gestational age - an italian cohort. Environ Int.

[29] Korten I, Ramsey K, Latzin P (2017). Air pollution during pregnancy and lung development in the child. Paediatr Respir Rev.

[30] Proietti E (2013). Air pollution during pregnancy and neonatal outcome: a review. J Aerosol Med Pulm Drug Deliv.

[31] Feldstein LR (2020). Multisystem inflammatory syndrome in U.S. children and adolescents. N Engl J Med.

