# Supplementary material for: Disease burden and attributable risk factors of neonatal disorders and their specific causes in China from 1990 to 2019 and its prediction to 2024
Source: BMC Public Health. 2023 Jan 18;23:122. doi: 10.1186/s12889-023-15050-x (PMC9845098; doi:10.1186/s12889-023-15050-x)
Supplement: Supplementary file 1 — Additional file 1: Table S1. All-age number and age-standardized rate of all measures for neonatal preterm birth and percentage changes by gender in China, 1990 and 2019. Table S2. All-age number and age-standardized rate of all measures for neonatal encephalopathy due to birth asphyxia and trauma and percentage changes by gender in China, 1990 and 2019. Table S3. All-age number and age-standardized rate of all measures for neonatal sepsis and other neonatal infections and percentage changes by gender in China, 1990 and 2019. Table S4. All-age number and age-standardized rate of all measures for hemolytic disease and other neonatal jaundice and percentage changes by gender in China, 1990 and 2019. Table S5. Prediction of age-standardized rate (per 100,000) of all six measures for neonatal preterm birth for the next 5 years according to ARIMA models with 95% confidence interval in China. Table S6. Prediction of age-standardized rate (per 100,000) of all six measures for neonatal encephalopathy due to birth asphyxia and trauma for the next 5 years according to ARIMA models with 95% confidence interval in China. Table S7. Prediction of age-standardized rate (per 100,000) of all six measures for neonatal sepsis and other neonatal infections for the next 5 years according to ARIMA models with 95% confidence interval in China. Table S8. Prediction of age-standardized rate (per 100,000) of all six measures for hemolytic disease and other neonatal jaundice for the next 5 years according to ARIMA models with 95% confidence interval in China. Table S9. ARIMA model parameters and their corresponding AIC and BIC for prediction of age-standardized rate (per 100,000) of all six measures for neonatal disorders for the next 5 years in China. Table S10. Points estimated and 95% uncertainty interval of attributable number and age-standardized rate of risk factors for neonatal preterm birth by gender in China, 2019. Table S11. Points estimated and 95% uncertainty interval of attributa [file 12889_2023_15050_MOESM1_ESM.docx]

**Supplementary material**

**Table S1** All-age number and age-standardized rate of all measures for neonatal preterm birth and percentage changes by gender in China, 1990 and 2019

| **Measures** | **All-age number in thousands (95% UI)** | | | **Age-standardized rate, per 100,000 (95% UI)** | | |
| --- | --- | --- | --- | --- | --- | --- |
|  | **1990** | **2019** | **change, %** | **1990** | **2019** | **change, %** |
| Deaths |  |  |  |  |  |  |
| Total | 157.3 (137.7, 178.2) | 22.1 (18.9, 25.7) | -85.9 (-88.4, -82.8) | 13.6 (11.9, 15.4) | 3.0 (2.6, 3.5) | -77.4 (-81.4, -72.6) |
| Male | 89.1 (77.1, 102.3) | 12.9 (10.7, 15.5) | -85.4 (-88.4, -81.8) | 14.5 (12.6, 16.7) | 2.7 (2.3, 3.1) | -77.1 (-81.8, -71.4) |
| Female | 68.2 (59.5, 77.0) | 9.2 (8.0, 10.5) | -86.5 (-88.7, -83.7) | 12.5 (10.9, 14.2) | 5.6 (4.8, 6.3) | -78.0 (-81.7, -73.5) |
| Incidence |  |  |  |  |  |  |
| Total | 1815.0 (1785.9, 1846.7) | 945.4 (930.1, 961.1) | -47.9 (-49.1, -46.7) | 157.4 (154.9, 160.2) | 132.0 (129.8, 134.2) | -16.1 (-18.1, -14.2) |
| Male | 981.7 (958.8, 1005.5) | 506.6 (494.8, 518.9) | -48.3 (-50.0, -46.6) | 160.5 (156.7, 164.4) | 131.3 (128.2, 134.4) | -18.2 (-20.8, -15.4) |
| Female | 833.3 (814.0, 852.9) | 438.7 (427.9, 449.1) | -47.3 (-49.2, -45.4) | 154.0 (150.4, 157.6) | 132.8 (129.5, 135.9) | -13.7 (-16.7, -10.7) |
| Prevalence |  |  |  |  |  |  |
| Total | 6303.5 (5541.5, 7079.8) | 8164.8 (7238.0, 9192.8) | 29.5 (18.1, 40.4) | 527.1 (463.5, 591.8) | 642.0 (575.4, 717.5) | 21.8 (11.4, 31.5) |
| Male | 3264.8 (3704.4, 2845.5) | 4113.8 (3623.5, 4672.2) | 26.0 (12.8, 40.5) | 524.9 (457.6, 595.0) | 632.1 (562.8, 710.9) | 20.4 (8.4, 33.4) |
| Female | 3038.7 (2662.8, 3457.6) | 4051.0 (3578.9, 4572.2) | 33.3 (19.8, 46.3) | 529.5 (464.8, 601.4) | 652.2 (583.6, 729.4) | 23.1 (10.7, 34.9) |
| DALYs |  |  |  |  |  |  |
| Total | 14688.5 (12916.3, 16535.8) | 3088.1 (2721.0, 3508.7) | -78.9 (-82.1, -75.2) | 1268.7 (1115.9, 1429.6) | 357.6 (313.9, 406.9) | -71.8 (-76.1, -66.6) |
| Male | 8277.0 (7190.2, 9419.4) | 1701.8 (1478.3, 1954.8) | -79.4 (-82.9, -75.1) | 1349.6 (1172.0, 1535.7) | 378.3 (326.6, 434.8) | -71.9 (-76.8, -66.1) |
| Female | 6411.5 (5624.9, 7189.8) | 1386.3 (1216.1, 1575.9) | -78.3 (-81.5, -74.5) | 1177.0 (1032.5, 1318.7) | 333.0 (296.2, 374.9) | -71.7 (-75.8, -66.8) |
| YLDs |  |  |  |  |  |  |
| Total | 727.4 (548.9, 937.8) | 1123.1 (873.3, 1403.9) | 54.3 (31.9, 78.7) | 59.0 (44.6, 76.2) | 85.2 (66.4, 106.3) | 44.5 (23.5, 66.7) |
| Male | 365.0 (273.6, 468.5) | 552.2 (431.4, 692.0) | 51.2 (29.2, 74.9) | 56.9 (42.9, 73.0) | 82.7 (64.2, 103.2) | 45.1 (24.6, 67.7) |
| Female | 362.3 (272.5, 468.4) | 570.8 (444.5, 718.0) | 57.5 (32.9, 82.8) | 61.1 (46.0, 79.1) | 87.8 (68.0, 109.8) | 43.6 (21.9, 66.1) |
| YLLs |  |  |  |  |  |  |
| Total | 13961.1 (12228.2, 15815) | 1965.0 (1679.8, 2283.7) | -85.9 (-88.4, -82.8) | 1209.7 (1059.6, 1370.2) | 272.4 (232.9, 316.3) | -77.4 (-81.4, -72.6) |
| Male | 7912.0 (6847.3, 9077.3) | 1149.5 (952.5, 1375.3) | -85.4 (-88.4, -81.8) | 1292.7 (1118.7, 1482.9) | 295.6 (245.0, 353.6) | -77.1 (-81.8, -71.4) |
| Female | 6049.0 (5279.1, 6832.4) | 815.5 (707.1, 933.6) | -86.5 (-88.7, -83.7) | 1115.9 (974.0, 1260.2) | 245.2 (212.6, 280.6) | -78.0 (-81.7, -73.5) |

Abbreviation: UI, uncertainty interval; DALYs, disability-adjusted life years; YLDs, years lived with disability; YLLs, years of life lost.

**Table S2** All-age number and age-standardized rate of all measures for neonatal encephalopathy due to birth asphyxia and trauma and percentage changes by gender in China, 1990 and 2019

| **Measures** | **All-age number in thousands (95% UI)** | | | **Age-standardized rate, per 100,000 (95% UI)** | | |
| --- | --- | --- | --- | --- | --- | --- |
|  | **1990** | **2019** | **change, %** | **1990** | **2019** | **change, %** |
| Deaths |  |  |  |  |  |  |
| Total | 93.5 (81.9, 108.9) | 14.5 (12.3, 17) | -84.4 (-87.3, -81.0) | 8.1 (7.1, 9.4) | 2.0 (1.7, 2.3) | -75.1 (-79.7, -69.7) |
| Male | 53.9 (45.8, 64.1) | 8.4 (6.9, 10.2) | -84.3 (-87.6, -80.4) | 8.8 (7.4, 10.4) | 2.1 (1.7, 2.6) | -75.3 (-80.5, -69.2) |
| Female | 39.5 (34.5, 46.2) | 6.0 (5.1, 7.0) | -84.7 (-87.3, -81.5) | 7.3 (6.3, 8.5) | 1.8 (1.5, 2.1) | -75.1 (-79.3, -69.9) |
| Incidence |  |  |  |  |  |  |
| Total | 107.9 (51.6, 214.8) | 24.9 (14.3, 45.2) | -76.8 (-81.6, -69.8) | 9.3 (4.4, 18.6) | 3.4 (2.0, 6.3) | -62.7 (-70.3, -51.4) |
| Male | 61.7 (29.8, 125.2) | 14.5 (8.3, 26.2) | -76.5 (-81.4, -68.9) | 10.0 (4.8, 20.4) | 3.1 (1.8, 5.7) | -62.7 (-70.6, -50.8) |
| Female | 46.1 (21.8, 89.8) | 10.4 (6.0, 18.9) | -77.2 (-82.0, -71.0) | 8.5 (4.0, 16.5) | 3.4 (2.0, 6.3) | -62.8 (-70.5, -52.6) |
| Prevalence |  |  |  |  |  |  |
| Total | 254.5 (102.8, 537) | 598.3 (428.7, 803.9) | 135.0 (36.8, 395.8) | 20.7 (8.5, 43.4) | 44.7 (31.9, 60.3) | 115.2 (25.2, 350.9) |
| Male | 130.0 (49.6, 276.6) | 330.3 (238.4, 441.3) | 154.0 (42.8, 479.5) | 20.4 (7.9, 43.2) | 48.2 (34.7, 64.4) | 136.2 (33.9, 434.8) |
| Female | 124.5 (52.3, 258.4) | 268.0 (188.8, 365.4) | 115.2 (26.0, 340.3) | 21.1 (8.9, 43.8) | 40.8 (28.7, 55.8) | 92.8 (13.9, 287.5) |
| DALYs |  |  |  |  |  |  |
| Total | 8405.0 (7385.9, 9766.5) | 1473.7 (1261.3, 1715.3) | -82.4 (-85.4, -78.5) | 728.2 (640, 845.7) | 192.5 (163.9, 225.5) | -73.5 (-78.2, -67.9) |
| Male | 4844.0 (4115.2, 5755.1) | 855.4 (718.1, 1020.3) | -82.3 (-85.8, -78.0) | 791.4 (672.4, 940.1) | 208.6 (173.2, 249.1) | -73.6 (-79.0, -67.2) |
| Female | 3561.0 (3118.6, 4144.5) | 618.3 (534.6, 709.0) | -82.6 (-85.5, -79.0) | 656.8 (575.0, 764.8) | 173.8 (149.8, 200.4) | -73.5 (-77.9, -68.1) |
| YLDs |  |  |  |  |  |  |
| Total | 94.4 (26.1, 234.2) | 184.8 (112.6, 277.7) | 95.6 (-3.1, 469.7) | 7.6 (2.1, 18.9) | 13.8 (8.5, 20.8) | 81.0 (-10.7, 425.7) |
| Male | 47.9 (11.9, 120.9) | 81.3 (49.1, 124.6) | 115.5 (4.6, 592.4) | 7.5 (1.8, 18.9) | 15.1 (9.4, 22.7) | 102.1 (-2.2, 546.8) |
| Female | 46.4 (14.1, 114.6) | 570.8 (444.5, 718.0) | 75.1 (-10.3, 357.3) | 7.8 (2.4, 19.3) | 12.4 (7.5, 19.1) | 58.9 (-18.7, 307.4) |
| YLLs |  |  |  |  |  |  |
| Total | 8310.6 (7282.8, 9676.3) | 1288.9 (1092.6, 1515.2) | -84.4 (-87.3, -81.0) | 720.6 (631.4, 839.1) | 178.6 (151.3, 209.9) | -75.2 (-79.7, -69.7) |
| Male | 4796.0 (4069.6, 5701.8) | 752.0 (616.5, 909.6) | -84.3 (-87.6, -80.4) | 783.9 (665.3, 932.1) | 193.4 (158.7, 234.2) | -75.3 (-80.5, -69.2) |
| Female | 3514.5 (3068.2, 4109.1) | 536.9 (461.2, 622.9) | -84.7 (-87.3, -81.5) | 648.9 (566.5, 758.7) | 161.3 (138.6, 187.3) | -75.1 (-79.3, -69.9) |

Abbreviation: UI, uncertainty interval; DALYs, disability-adjusted life years; YLDs, years lived with disability; YLLs, years of life lost.

**Table S3** All-age number and age-standardized rate of all measures for neonatal sepsis and other neonatal infections and percentage changes by gender in China, 1990 and 2019

| **Measures** | **All-age number in thousands (95% UI)** | | | **Age-standardized rate, per 100,000 (95% UI)** | | |
| --- | --- | --- | --- | --- | --- | --- |
|  | **1990** | **2019** | **change, %** | **1990** | **2019** | **change, %** |
| Deaths |  |  |  |  |  |  |
| Total | 4.8 (3.7, 5.6) | 1.9 (1.5, 2.2) | -59.9 (-68.8, -45.3) | 0.4 (0.3, 0.5) | 0.2 (0.2, 0.3) | -36.4 (-50.5, -13.0) |
| Male | 3.0 (2.3, 3.6) | 1.1 (0.9, 1.4) | -61.6 (-71.6, -45.7) | 0.5 (0.4, 0.6) | 0.3 (0.2, 0.4) | -40.0 (-55.6, -15.0) |
| Female | 1.7 (1.3, 2.0) | 0.7 (0.6, 0.8) | -57.0 (-67.4, -42.2) | 0.3 (0.3, 0.4) | 0.2 (0.2, 0.3) | -30.5 (-47.3, -6.4) |
| Incidence |  |  |  |  |  |  |
| Total | 1045.0 (698.4, 1473.5) | 1106.4 (742.9, 1537.5) | 5.8 (-6.2, 18.4) | 90.6 (60.5, 127.7) | 154.1 (103.6, 214.2) | 70.1 (50.5, 90.4) |
| Male | 575.4 (384.4, 814.6) | 590.2 (395.5, 826.1) | 2.5 (-8.9, 14.2) | 94.0 (62.8, 133.1) | 152.6 (102.1, 213.4) | 62.3 (43.9, 80.9) |
| Female | 469.6 (313.6, 663.0) | 516.1 (347.4, 718.2) | 9.8 (-4.2, 24.4) | 86.7 (57.9, 122.4) | 155.9 (105.0, 216.6) | 79.8 (56.6, 103.9) |
| Prevalence |  |  |  |  |  |  |
| Total | 1664.8 (1169.3, 2270.5) | 7135.9 (5391.9, 9338.1) | 328.6 (245.1, 454.5) | 138.3 (97.7, 188.2) | 528.5 (400.0, 690.1) | 281.9 (206.4, 395.9) |
| Male | 877.2 (614.3, 1196.7) | 3473.5 (2634.1, 4510.6) | 295.9 (219.6, 414.8) | 140.4 (98.5, 190.5) | 505.0 (384.0, 655.6) | 259.5 (191.2, 366.4) |
| Female | 787.6 (555.6, 1061.3) | 3662.3 (2753.0, 4819.3) | 364.9 (269.7, 501.8) | 136.0 (96.2, 182.4) | 553.1 (418.3, 724.7) | 306.4 (223.5, 424.1) |
| DALYs |  |  |  |  |  |  |
| Total | 617.8 (466.3, 841.6) | 1749.6 (1221.9, 2407.4) | 183.1 (106.8, 286.4) | 52.4 (39.8, 70.4) | 140.5 (100.8, 190.2) | 167.7 (99.4, 263.6) |
| Male | 370.6 (280.1, 495.1) | 874.4 (611.7, 1207.6) | 135.9 (71.2, 229.5) | 59.8 (45.4, 79.1) | 138.5 (100.0, 187.0) | 131.6 (71.5, 220.7) |
| Female | 247.1 (182.9, 346.3) | 875.1 (602.6, 1205.0) | 254.0 (161.3, 385.2) | 44.1 (33.1, 60.8) | 142.1 (101.5, 192.5) | 221.7 (139.5, 334.2) |
| YLDs |  |  |  |  |  |  |
| Total | 191.7 (75.6, 416.3) | 1579.2 (1045.0, 2232.6) | 723.7 (370.9, 1775.7) | 15.5 (6.1, 33.8) | 117.0 (77.2, 166.3) | 651.6 (330.1, 1585.5) |
| Male | 101.1 (38.6, 220.4) | 771.2 (509.6, 1101.2) | 662.4 (326.4, 1661.5) | 15.7 (6.1, 34.3) | 112.1 (74.5, 159.9) | 610.4 (297.4, 1533.6) |
| Female | 90.5 (36.3, 190.7) | 808.0 (539.5, 1144.1) | 792.0 (417.2, 1868.0) | 15.3 (6.2, 31.9) | 122.1 (80.6, 172.6) | 696.9 (364.6, 1642.7) |
| YLLs |  |  |  |  |  |  |
| Total | 426.1 (336.7, 500.1) | 170.4 (141.8, 200.6) | -60.0 (-68.9, -45.3) | 36.9 (29.1, 43.3) | 23.4 (19.4, 27.6) | -36.4 (-50.6, -13.1) |
| Male | 269.5 (210.2, 323.9) | 103.2 (82.3, 124.6) | -61.6 (-71.6, -45.7) | 44.0 (34.3, 52.9) | 26.3 (21.0, 31.8) | -40.0 (-55.7, -15.1) |
| Female | 156.5 (124, 186.2) | 67.1 (57.0, 78.3) | -57.1 (-67.4, -42.2) | 28.8 (22.8, 34.3) | 20.0 (17.0, 23.3) | -30.5 (-47.3, -6.4) |

Abbreviation: UI, uncertainty interval; DALYs, disability-adjusted life years; YLDs, years lived with disability; YLLs, years of life lost.

**Table S4** All-age number and age-standardized rate of all measures for hemolytic disease and other neonatal jaundice and percentage changes by gender in China, 1990 and 2019

| **Measures** | **All-age number in thousands (95% UI)** | | | **Age-standardized rate, per 100,000 (95% UI)** | | |
| --- | --- | --- | --- | --- | --- | --- |
|  | **1990** | **2019** | **change, %** | **1990** | **2019** | **change, %** |
| Deaths |  |  |  |  |  |  |
| Total | 8.8 (7.3, 10.4) | 1.0 (0.8, 1.1) | -89.2 (-91.7, -85.8) | 0.8 (0.6, 0.9) | 0.1 (0.1, 0.2) | -82.9 (-86.8, -77.5) |
| Male | 4.7 (3.9, 5.6) | 0.6 (0.5, 0.7) | -87.7 (-90.9, -84.1) | 0.8 (0.6, 0.9) | 0.1 (0.1, 0.2) | -80.9 (-85.8, -75.2) |
| Female | 4.0 (3.2, 4.9) | 0.4 (0.3, 0.4) | -90.8 (-93.3, -87.3) | 0.7 (0.6, 0.9) | 0.1 (0.1, 0.1) | -85.3 (-89.3, -79.6) |
| Incidence |  |  |  |  |  |  |
| Total | 23.4 (13.2, 45.8) | 8.8 (3.4, 22.4) | -62.0 (-75.8, -49.6) | 2.0 (1.2, 4.0) | 1.2 (0.5, 3.1) | -38.8 (-61.1, -18.9) |
| Male | 12.7 (7.3, 24.6) | 5.6 (2.6, 12.8) | -55.7 (-66.4, -46.2) | 2.1 (1.2, 4.0) | 1.5 (0.7, 3.3) | -29.9 (-46.8, -14.8) |
| Female | 10.6 (5.9, 21.1) | 3.2 (0.8, 9.5) | -69.4 (-87.6, -53.6) | 2.0 (1.1, 3.9) | 1.0 (0.3, 2.9) | -50.0 (-79.7, -24.0) |
| Prevalence |  |  |  |  |  |  |
| Total | 97.9 (85.2, 111.9) | 163.5 (146.1, 181.5) | 66.9 (55.9, 78.9) | 7.9 (6.9, 9.1) | 12.2 (10.9, 13.5) | 53.1 (43.0, 64.1) |
| Male | 51.1 (44.2, 58.6) | 85.6 (76.2, 95.1) | 67.4 (56.4, 79.2) | 8.0 (6.9, 9.1) | 12.6 (11.2, 14.0) | 57.1 (47.0, 68.1) |
| Female | 46.7 (40.8, 53.5) | 77.8 (70.0, 86.6) | 66.4 (55.1, 79.1) | 7.9 (6.9, 9.0) | 11.8 (10.6, 13.1) | 48.5 (38.3, 59.7) |
| DALYs |  |  |  |  |  |  |
| Total | 824.0 (687.5, 971.1) | 144.8 (123.9, 167.4) | -82.4 (-85.7, -78.2) | 71.1 (59.3, 83.7) | 16.0 (13.7, 18.5) | -77.3 (-81.6, -71.5) |
| Male | 378.5 (303.9, 458.6) | 83.9 (71.4, 97.7) | -81.1 (-85.0, -76.6) | 72.5 (61.0, 86.4) | 17.8 (15.1, 20.9) | -75.3 (-80.5, -69.0) |
| Female | 247.1 (182.9, 346.3) | 60.8 (51.7, 70.1) | -83.9 (-87.6, -78.8) | 69.4 (55.6, 84.1) | 13.9 (11.9, 16.1) | -79.8 (-84.4, -73.0) |
| YLDs |  |  |  |  |  |  |
| Total | 42.7 (32.1, 54.3) | 60.5 (45.6, 76.2) | 41.5 (23.0, 59.8) | 3.4 (2.6, 4.4) | 4.5 (3.4, 5.6) | 30.1 (13.5, 46.5) |
| Male | 22.3 (16.7, 28.4) | 32.3 (24.4, 40.4) | 44.5 (26.6, 62.0) | 3.5 (2.6, 4.4) | 4.7 (3.6, 5.9) | 35.4 (18.3, 52.4) |
| Female | 20.4 (15.4, 25.9) | 28.2 (21.2, 35.5) | 38.2 (18.5, 57.0) | 3.4 (2.6, 4.4) | 4.2 (3.2, 5.4) | 24.1 (7.0, 40.7) |
| YLLs |  |  |  |  |  |  |
| Total | 781.3 (648.9, 925.0) | 84.3 (69.4, 100.6) | -89.2 (-91.7, -85.8) | 67.6 (56.1, 80.0) | 11.5 (9.5, 13.7) | -82.9 (-86.8, -77.6) |
| Male | 423.1 (352.4, 505.0) | 51.6 (41.6, 63.5) | -87.7 (-90.9, -84.1) | 69.0 (57.5, 82.4) | 13.1 (10.6, 16.1) | -80.9 (-85.8, -75.2) |
| Female | 358.1 (284.9, 438.5) | 32.6 (27.0, 38.9) | -90.8 (-93.3, -87.3) | 65.9 (52.4, 80.7) | 9.6 (8.0, 11.5) | -85.3 (-89.3, -79.6) |

Abbreviation: UI, uncertainty interval; DALYs, disability-adjusted life years; YLDs, years lived with disability; YLLs, years of life lost.

**Table S5** Prediction of age-standardized rate (per 100,000) of all six measures for neonatal preterm birth for the next 5 years according to ARIMA models with 95% confidence interval in China

| **Measures** | **2020** | **2021** | **2022** | **2023** | **2024** |
| --- | --- | --- | --- | --- | --- |
| Deaths |  |  |  |  |  |
| Both | 2.8 (2.4, 3.2) | 2.6 (1.7, 3.5) | 2.4 (1.0, 3.9) | 2.3 (0.3, 4.3) | 2.2 (-0.3, 4.8) |
| Male | 3.0 (2.5, 3.4) | 2.6 (1.7, 3.5) | 2.2 (1.0, 3.4) | 1.8 (0.4, 3.3) | 1.5 (-0.1, 3.1) |
| Female | 2.5 (2.1, 2.9) | 2.3 (1.5, 3.2) | 2.2 (0.8, 3.6) | 2.1 (0.1, 4.0) | 2.0 (-0.4, 4.4) |
| Incidence |  |  |  |  |  |
| Both | 131.2 (126.6, 135.7) | 129.9 (121.6, 138.1) | 129.0 (119.3, 138.6) | 128.0 (117.2, 138.9) | 127.1 (115.1, 139.1) |
| Male | 130.4 (125.8, 135.0) | 129.0 (120.5, 137.5) | 127.9 (118.0, 137.9) | 126.9 (115.6, 138.2) | 125.9 (113.4, 138.4) |
| Female | 132.2 (126.9, 137.6) | 131.7 (124.0, 139.5) | 131.2 (121.4, 141.0) | 130.7 (119.1, 142.4) | 130.2 (116.8, 143.6) |
| Prevalence |  |  |  |  |  |
| Both | 649.2 (647.5, 650.9) | 656.2 (651.7, 660.6) | 663.2 (655.7, 670.7) | 670.2 (659.3, 681.1) | 677.2 (662.5, 691.9) |
| Male | 640.7 (638.8, 642.6) | 649.0 (644.0, 654.1) | 657.4 (648.9, 665.9) | 665.7 (653.4, 678.1) | 674.1 (657.5, 690.7) |
| Female | 658.1 (656.6, 659.6) | 664.0 (659.7, 668.3) | 669.9 (662.1, 677.6) | 675.8 (663.9, 687.6) | 681.7 (665.2, 698.1) |
| DALYs |  |  |  |  |  |
| Both | 330.8 (293.2, 368.4) | 300.5 (223.6, 377.3) | 270.1 (168.1, 372.1) | 239.8 (117.7, 361.9) | 209.5 (70.2, 348.8) |
| Male | 349.9 (310.7, 389.2) | 317.6 (237.8, 397.4) | 285.2 (179.4, 391.0) | 252.8 (126.3, 379.4) | 220.5 (76.1, 364.9) |
| Female | 307.7 (272.0, 343.5) | 279.7 (205.9, 353.4) | 251.6 (153.6, 349.5) | 223.5 (106.2, 340.8) | 195.4 (61.5, 329.2) |
| YLDs |  |  |  |  |  |
| Both | 85.4 (85.1, 85.7) | 85.6 (84.8, 86.4) | 85.8 (84.3, 87.2) | 85.9 (83.7, 88.2) | 86.1 (83.0, 89.2) |
| Male | 82.9 (82.7, 83.2) | 83.2 (82.4, 84.0) | 83.5 (82.0, 84.9) | 83.7 (81.5, 86.0) | 84.0 (80.8, 87.2) |
| Female | 87.9 (87.6, 88.3) | 88.0 (87.0, 89.1) | 88.2 (86.3, 90.0) | 88.3 (85.4, 91.2) | 88.4 (84.4, 92.4) |
| YLLs |  |  |  |  |  |
| Both | 252.8 (214.8, 290.9) | 235.8 (155.0, 316.6) | 221.0 (92.3, 349.7) | 208.1 (28.4, 387.8) | 196.8 (-35.5, 429.3) |
| Male | 266.6 (227.1, 306.1) | 233.4 (153.0, 313.7) | 200.2 (93.6, 306.7) | 166.9 (39.4, 294.4) | 133.7 (-11.6, 279.1) |
| Female | 227.7 (191.5, 263.9) | 212.5 (135.8, 289.3) | 199.3 (77.2, 321.5) | 187.8 (17.4, 358.3) | 177.8 (-42.3, 398.1) |

Abbreviation: DALYs, disability-adjusted life years; YLDs, years lived with disability; YLLs, years of life lost.

**Table S6** Prediction of age-standardized rate (per 100,000) of all six measures for neonatal encephalopathy due to birth asphyxia and trauma for the next 5 years according to ARIMA models with 95% confidence interval in China

| **Measures** | **2020** | **2021** | **2022** | **2023** | **2024** |
| --- | --- | --- | --- | --- | --- |
| Deaths |  |  |  |  |  |
| Both | 1.8 (1.5, 2.1) | 1.7 (1.0, 2.4) | 1.6 (0.4, 2.8) | 1.4 (-0.2, 3.2) | 1.3 (-1.0, 3.7) |
| Male | 2.0 (1.6, 2.3) | 1.8 (1.0, 2.6) | 1.7 (0.3, 3.0) | 1.5 (-0.3, 3.5) | 1.4 (-1.2, 4.0) |
| Female | 1.6 (1.4, 1.9) | 1.5 (0.9, 2.1) | 1.4 (0.4, 2.4) | 1.3 (-0.2, 2.8) | 1.2 (-0.8, 3.2) |
| Incidence |  |  |  |  |  |
| Both | 3.3 (3.0, 3.5) | 3.1 (2.5, 3.6) | 2.9 (2.1, 3.6) | 2.7 (1.7, 3.6) | 2.5 (1.4, 3.5) |
| Male | 3.5 (3.2, 3.8) | 3.3 (2.6, 3.9) | 3.0 (2.2, 3.9) | 2.8 (1.8, 3.8) | 2.6 (1.5, 3.7) |
| Female | 3.0 (2.8, 3.2) | 2.8 (2.3, 3.4) | 2.6 (1.9, 3.3) | 2.5 (1.6, 3.3) | 2.3 (1.3, 3.3) |
| Prevalence |  |  |  |  |  |
| Both | 45.2 (44.9, 45.4) | 45.7 (45.0, 46.3) | 46.2 (45.0, 47.5) | 46.7 (44.7, 48.7) | 47.2 (44.5, 49.9) |
| Male | 48.7 (48.3, 49.0) | 49.0 (47.8, 50.2) | 49.3 (46.8, 51.8) | 49.6 (45.5, 53.8) | 49.9 (44.0, 55.9) |
| Female | 41.0 (40.6, 41.3) | 41.2 (40.2, 42.2) | 41.4 (39.6, 43.2) | 41.6 (38.9, 44.3) | 41.8 (38.2, 45.4) |
| DALYs |  |  |  |  |  |
| Both | 180.3 (151.8, 208.8) | 168.1 (104.3, 231.8) | 155.8 (49.2, 262.5) | 143.6 (-12.4, 299.8) | 131.4 (-79.9, 342.9) |
| Male | 195.4 (163.7, 227.1) | 182.2 (111.3, 253.2) | 169.1 (50.4, 287.8) | 155.9 (-17.8, 329.7) | 142.8 (-92.5, 378.1) |
| Female | 162.7 (137.8, 187.6) | 151.6 (95.9, 207.3) | 140.5 (47.3, 233.7) | 129.4 (-6.9, 265.8) | 118.3 (-66.3, 303.0) |
| YLDs |  |  |  |  |  |
| Both | 13.7 (13.7, 13.8) | 13.6 (13.3, 13.8) | 13.4 (12.9, 13.9) | 13.3 (12.5, 14.0) | 13.1 (12.0, 14.3) |
| Male | 15.2 (15.0, 15.3) | 15.3 (14.9, 15.8) | 15.5 (14.7, 16.4) | 15.8 (14.6, 17.1) | 16.1 (14.6, 17.7) |
| Female | 12.2 (12.1, 12.3) | 12.0 (11.7, 12.4) | 11.8 (11.1, 12.6) | 11.6 (10.4, 12.9) | 11.4 (9.6, 13.2) |
| YLLs |  |  |  |  |  |
| Both | 166.5 (138.0, 194.9) | 154.3 (90.7, 218) | 142.2 (35.6, 248.7) | 130.1 (-25.8, 286.0) | 117.9 (-93.2, 329.1) |
| Male | 180.2 (148.5, 211.9) | 167.0 (96.1, 237.8) | 153.8 (35.2, 272.3) | 140.6 (-32.9, 314.1) | 127.4 (-107.5, 362.3) |
| Female | 150.4 (125.5, 175.3) | 139.5 (83.9, 195.2) | 128.7 (35.6, 221.8) | 117.8 (-18.4, 254.1) | 106.9 (-77.5, 291.4) |

Abbreviation: DALYs, disability-adjusted life years; YLDs, years lived with disability; YLLs, years of life lost.

**Table S7** Prediction of age-standardized rate (per 100,000) of all six measures for neonatal sepsis and other neonatal infections for the next 5 years according to ARIMA models with 95% confidence interval in China

| **Measures** | **2020** | **2021** | **2022** | **2023** | **2024** |
| --- | --- | --- | --- | --- | --- |
| Deaths |  |  |  |  |  |
| Both | 0.2 (0.2, 0.2) | 0.2 (0.2, 0.2) | 0.2 (0.1, 0.2) | 0.2 (0.1, 0.2) | 0.2 (0.1, 0.3) |
| Male | 0.2 (0.2, 0.3) | 0.2 (0.2, 0.3) | 0.2 (0.2, 0.3) | 0.2 (0.2, 0.3) | 0.2 (0.1, 0.3) |
| Female | 0.2 (0.2, 0.2) | 0.2 (0.1, 0.2) | 0.2 (0.1, 0.2) | 0.2 (0.1, 0.2) | 0.1 (0.1, 0.2) |
| Incidence |  |  |  |  |  |
| Both | 160.9 (159.8, 162.0) | 167.8 (164.0, 171.6) | 174.6 (166.9, 182.4) | 181.5 (169.0, 194.0) | 188.4 (170.5, 206.3) |
| Male | 158.1 (156.9, 159.4) | 161.5 (157.7, 165.3) | 163.2 (156.1, 170.2) | 165.0 (154.9, 175.1) | 168.3 (155.4, 181.2) |
| Female | 163.1 (162.0, 164.1) | 170.3 (166.7, 173.9) | 177.6 (170.3, 184.9) | 184.8 (173.0, 196.6) | 192.1 (175.1, 209.0) |
| Prevalence |  |  |  |  |  |
| Both | 547.3 (542.1, 552.5) | 566.2 (546.7, 585.7) | 585.1 (543.5, 626.6) | 603.9 (535.4, 672.4) | 622.8 (523.2, 722.4) |
| Male | 519.7 (514.8, 524.5) | 534.3 (516.2, 552.3) | 548.9 (510.6, 587.1) | 563.5 (500.5, 626.4) | 578.0 (486.6, 669.5) |
| Female | 573.4 (567.8, 579.0) | 586.1 (566.2, 606.0) | 593.7 (553.3, 634.0) | 602.2 (539.7, 664.8) | 614.9 (530.6, 699.2) |
| DALYs |  |  |  |  |  |
| Both | 141.9 (139.6, 144.1) | 143.2 (135.9, 150.5) | 144.6 (130.5, 158.6) | 145.9 (123.8, 168.0) | 147.3 (116.0, 178.5) |
| Male | 139.8 (136.8, 142.7) | 141.0 (132.8, 149.2) | 142.3 (127.4, 157.1) | 143.5 (120.9, 166.1) | 144.8 (113.4, 176.1) |
| Female | 144.3 (141.5, 147.1) | 146.4 (138.8, 154.1) | 148.6 (134.8, 162.4) | 150.7 (129.8, 171.7) | 152.9 (123.8, 181.9) |
| YLDs |  |  |  |  |  |
| Both | 118.6 (116.6, 120.6) | 114.6 (112.4, 124.7) | 110.0 (105.9, 129.3) | 105.3 (99.1, 134.6) | 101.4 (93.2, 140.6) |
| Male | 113.0 (111.1, 114.8) | 112.5 (106.2, 118.8) | 111.7 (99.2, 124.2) | 111.5 (92.1, 130.8) | 111.6 (85.2, 138.1) |
| Female | 124.6 (122.6, 126.6) | 127.1 (120.1, 134.1) | 129.5 (115.2, 143.8) | 132.0 (108.9, 155.2) | 134.5 (101.2, 167.8) |
| YLLs |  |  |  |  |  |
| Both | 22.5 (21.4, 23.6) | 21.6 (19.1, 24.1) | 20.7 (16.5, 24.9) | 19.8 (13.7, 26.0) | 18.9 (10.6, 27.3) |
| Male | 25.7 (24.3, 27.1) | 25.1 (22.6, 27.7) | 24.5 (20.7, 28.3) | 23.9 (18.8, 29.1) | 23.3 (16.7, 30.0) |
| Female | 19.5 (18.4, 20.6) | 19.0 (17.1, 21.0) | 18.6 (15.8, 21.4) | 18.1 (14.4, 21.8) | 17.6 (12.9, 22.4) |

Abbreviation: DALYs, disability-adjusted life years; YLDs, years lived with disability; YLLs, years of life lost.

**Table S8** Prediction of age-standardized rate (per 100,000) of all six measures for hemolytic disease and other neonatal jaundice for the next 5 years according to ARIMA models with 95% confidence interval in China

| **Measures** | **2020** | **2021** | **2022** | **2023** | **2024** |
| --- | --- | --- | --- | --- | --- |
| Deaths |  |  |  |  |  |
| Both | 0.1 (0, 0.1) | 0.1 (0, 0.1) | 0 (0, 0.1) | 0 (0, 0.1) | 0 (0, 0.1) |
| Male | 0.1 (0.1, 0.1) | 0.1 (0, 0.1) | 0 (0, 0.1) | 0 (0, 0.1) | 0 (0, 0.1) |
| Female | 0 (0, 0.1) | 0 (0, 0.1) | 0 (0, 0.1) | 0 (0, 0.2) | 0 (0, 0.2) |
| Incidence |  |  |  |  |  |
| Both | 1.1 (1.0, 1.2) | 1.1 (0.9, 1.2) | 1.0 (0.8, 1.2) | 1.0 (0.7, 1.2) | 0.9 (0.6, 1.2) |
| Male | 1.4 (1.3, 1.4) | 1.3 (1.2, 1.5) | 1.3 (1.1, 1.5) | 1.3 (1.1, 1.6) | 1.3 (1.0, 1.6) |
| Female | 0.9 (0.8, 0.9) | 0.8 (0.6, 0.9) | 0.7 (0.5, 0.9) | 0.6 (0.3, 0.9) | 0.5 (0.2, 0.8) |
| Prevalence |  |  |  |  |  |
| Both | 12.2 (12.1, 12.2) | 12.2 (12.1, 12.2) | 12.1 (12.0, 12.3) | 12.1 (11.9, 12.4) | 12.1 (11.7, 12.5) |
| Male | 12.5 (12.5, 12.6) | 12.5 (12.4, 12.6) | 12.5 (12.3, 12.7) | 12.5 (12.2, 12.8) | 12.5 (12.1, 12.9) |
| Female | 11.7 (11.7, 11.8) | 11.7 (11.6, 11.8) | 11.7 (11.5, 11.8) | 11.6 (11.4, 11.9) | 11.6 (11.3, 12.0) |
| DALYs |  |  |  |  |  |
| Both | 14.6 (12.8, 16.5) | 13.4 (10.1, 16.6) | 12.0 (7.2, 16.8) | 10.7 (3.7, 17.7) | 9.4 (0.3, 18.4) |
| Male | 16.3 (14.0, 18.6) | 14.8 (10.7, 18.9) | 13.3 (7.3, 19.2) | 11.7 (3.7, 19.8) | 10.2 (0, 20.5) |
| Female | 12.4 (9.7, 15.0) | 10.6 (6.2, 15.1) | 8.8 (3.0, 14.7) | 7.0 (0, 14.1) | 5.2 (-2.9, 13.3) |
| YLDs |  |  |  |  |  |
| Both | 4.5 (4.5, 4.5) | 4.5 (4.4, 4.5) | 4.5 (4.4, 4.5) | 4.5 (4.4, 4.6) | 4.4 (4.3, 4.6) |
| Male | 4.7 (4.7, 4.7) | 4.7 (4.6, 4.7) | 4.7 (4.6, 4.8) | 4.7 (4.5, 4.8) | 4.7 (4.5, 4.9) |
| Female | 4.2 (4.2, 4.2) | 4.2 (4.2, 4.2) | 4.2 (4.2, 4.2) | 4.2 (4.1, 4.3) | 4.2 (4.1, 4.3) |
| YLLs |  |  |  |  |  |
| Both | 10.1 (8.3, 11.9) | 8.8 (5.5, 12.1) | 7.5 (2.7, 12.3) | 6.2 (-0.7, 13.2) | 4.9 (-4.1, 14.0) |
| Male | 11.6 (9.3, 13.9) | 10.1 (6.0, 14.2) | 8.5 (2.5, 14.6) | 7.0 (-1.0, 15.1) | 5.5 (-4.8, 15.9) |
| Female | 8.7 (6.2, 11.3) | 8.1 (3.4, 12.8) | 7.4 (-0.7, 15.7) | 6.8 (-5.8, 19.5) | 6.2 (-11.5, 24.0) |

Abbreviation: DALYs, disability-adjusted life years; YLDs, years lived with disability; YLLs, years of life lost.

**Table S9** ARIMA model parameters and their corresponding AIC and BIC for prediction of age-standardized rate (per 100,000) of all six measures for neonatal disorders for the next 5 years in China

| **Measures** | **ND** | | | **NPB** | | | **NE** | | | **NS** | | | **HD** | | |
| --- | --- | --- | --- | --- | --- | --- | --- | --- | --- | --- | --- | --- | --- | --- | --- |
| Deaths | Parameters | AIC | BIC | Parameters | AIC | BIC | Parameters | AIC | BIC | Parameters | AIC | BIC | Parameters | AIC | BIC |
| Both | ARIMA (0,2,0) | 28.88 | 30.21 | ARIMA (1,1,0) | 1.47 | 1.27 | ARIMA (0,2,0) | -19.96 | -18.63 | ARIMA (0,2,0) | -200.86 | -199.52 | ARIMA (2,2,1) | -169.57 | -164.24 |
| Male | ARIMA (0,2,0) | 32.76 | 34.09 | ARIMA (0,1,1) | 1.10 | 5.20 | ARIMA (0,2,0) | -13.98 | -12.65 | ARIMA (0,2,1) | -186.83 | -184.17 | ARIMA (0,2,1) | -158.92 | -156.26 |
| Female | ARIMA (0,2,0) | 23.99 | 25.32 | ARIMA (1,1,0) | 4.42 | 1.69 | ARIMA (0,2,0) | -27.52 | -26.19 | ARIMA (0,2,1) | -200.81 | -198.15 | ARIMA (0,2,2) | -152.06 | -148.07 |
| Incidence |  |  |  |  |  |  |  |  |  |  |  |  |  |  |  |
| Both | ARIMA (0,2,2) | 147.41 | 151.41 | ARIMA (0,1,2) | 137.63 | 143.10 | ARIMA (0,1,1) | -28.37 | -24.27 | ARIMA (0,2,2) | 52.94 | 56.94 | ARIMA (0,2,1) | -90.37 | -87.70 |
| Male | ARIMA (0,2,1) | 150.77 | 153.44 | ARIMA (0,1,2) | 138.43 | 143.90 | ARIMA (0,1,1) | -24.43 | -20.33 | ARIMA (2,2,0) | 60.86 | 64.86 | ARIMA (0,1,1) | -104.03 | -99.93 |
| Female | ARIMA (0,2,2) | 147.34 | 151.34 | ARIMA (0,2,1) | 140.22 | 142.89 | ARIMA (0,1,1) | -32.66 | -28.56 | ARIMA (0,2,2) | 49.13 | 53.13 | ARIMA (0,2,1) | -85.41 | -82.75 |
| Prevalence |  |  |  |  |  |  |  |  |  |  |  |  |  |  |  |
| Both | ARIMA (0,3,1) | 144.18 | 146.77 | ARIMA (0,2,2) | 74.85 | 78.84 | ARIMA (2,2,0) | -40.61 | -36.62 | ARIMA (0,2,2) | 141.69 | 145.69 | ARIMA (1,2,1) | -163.63 | -159.64 |
| Male | ARIMA (0,3,1) | 145.75 | 148.34 | ARIMA (0,2,2) | 82.24 | 86.24 | ARIMA (2,2,1) | -14.52 | -9.20 | ARIMA (0,2,2) | 137.44 | 141.43 | ARIMA (1,2,1) | -155.60 | -151.60 |
| Female | ARIMA (0,3,1) | 149.27 | 151.87 | ARIMA (0,2,1) | 68.02 | 70.69 | ARIMA (1,1,1) | -10.90 | -6.79 | ARIMA (2,2,1) | 145.96 | 151.29 | ARIMA (1,2,1) | -172.45 | -168.45 |
| DALYs |  |  |  |  |  |  |  |  |  |  |  |  |  |  |  |
| Both | ARIMA (0,2,0) | 280.56 | 281.90 | ARIMA (0,1,1) | 258.49 | 262.59 | ARIMA (0,2,0) | 231.39 | 232.72 | ARIMA (0,2,2) | 93.71 | 97.71 | ARIMA (2,2,1) | 81.46 | 86.79 |
| Male | ARIMA (0,2,0) | 284.51 | 285.84 | ARIMA (0,1,1) | 260.98 | 265.08 | ARIMA (0,2,0) | 237.38 | 238.71 | ARIMA (0,2,1) | 106.07 | 108.73 | ARIMA (0,2,1) | 92.13 | 94.79 |
| Female | ARIMA (0,2,0) | 275.59 | 276.92 | ARIMA (0,1,1) | 255.68 | 259.78 | ARIMA (0,2,0) | 223.81 | 225.14 | ARIMA (0,2,1) | 102.59 | 105.26 | ARIMA (1,1,0) | 103.63 | 107.73 |
| YLDs |  |  |  |  |  |  |  |  |  |  |  |  |  |  |  |
| Both | ARIMA (1,1,2) | 89.57 | 96.40 | ARIMA (1,1,1) | -22.02 | -17.92 | ARIMA (4,1,0) | -102.76 | -95.92 | ARIMA (2,2,0) | 85.45 | 89.45 | ARIMA (0,2,1) | -204.37 | -201.71 |
| Male | ARIMA (1,1,2) | 94.07 | 100.91 | ARIMA (2,1,0) | -22.21 | -18.11 | ARIMA (2,1,1) | -61.41 | -54.58 | ARIMA (2,2,1) | 83.59 | 88.92 | ARIMA (0,2,1) | -178.36 | -175.70 |
| Female | ARIMA (2,1,1) | 88.44 | 95.28 | ARIMA (0,2,1) | -11.74 | -9.07 | ARIMA (0,2,2) | -76.61 | -72.61 | ARIMA (0,2,2) | 87.24 | 91.23 | ARIMA (1,2,1) | -244.04 | -240.04 |
| YLLs |  |  |  |  |  |  |  |  |  |  |  |  |  |  |  |
| Both | ARIMA (0,2,0) | 280.15 | 281.49 | ARIMA (1,1,0) | 258.77 | 261.5 | ARIMA (0,2,0) | 231.32 | 232.65 | ARIMA (0,2,0) | 50.4 | 51.74 | ARIMA (2,2,1) | 81.61 | 86.94 |
| Male | ARIMA (0,2,0) | 284.04 | 285.37 | ARIMA (0,1,1) | 261.33 | 265.43 | ARIMA (0,2,0) | 237.29 | 238.63 | ARIMA (0,2,1) | 64.43 | 67.09 | ARIMA (0,2,1) | 92.27 | 94.93 |
| Female | ARIMA (0,2,0) | 275.26 | 276.59 | ARIMA (1,1,0) | 255.81 | 258.55 | ARIMA (0,2,0) | 223.76 | 225.09 | ARIMA (0,2,1) | 50.42 | 53.08 | ARIMA (0,2,2) | 99.14 | 103.14 |

Abbreviation: ND, neonatal disorders; NPB, neonatal preterm birth; NE, neonatal encephalopathy due to birth asphyxia and trauma; NS, neonatal sepsis and other neonatal infections; HD, hemolytic disease and other neonatal jaundice; DALYs, disability-adjusted life years; YLDs, years lived with disability; YLLs, years of life lost.

**Table S10** Points estimated and 95% uncertainty interval of attributable number and age-standardized rate of risk factors for neonatal preterm birth by gender in China, 2019

| **Measures** | **All risk factors** | **Low birth weight** | **Short gestation** | **Ambient particulate matter pollution** | **Household air pollution from solid fuels** |  |
| --- | --- | --- | --- | --- | --- | --- |
|  |  |  |  |  |  |  |
| **Attributable number in thousands** | | | | | |  |
| Deaths |  |  |  |  |  |  |
| Both | 22.1 (18.9, 25.7) | 22.1 (18.9, 25.7) | 22.1 (18.9, 25.7) | 2.2 (1.6, 2.7) | 0.8 (0.5, 1.3) |  |
| Female | 9.1 (7.9, 10.5) | 9.1 (7.9, 10.5) | 9.1 (7.9, 10.5) | 0.9 (0.6, 1.2) | 0.3 (0.2, 0.5) |  |
| Male | 12.9 (10.7, 15.4) | 12.9 (10.7, 15.4) | 12.9 (10.7, 15.4) | 1.2 (0.9, 1.6) | 0.5 (0.3, 0.7) |  |
| DALYs |  |  |  |  |  |  |
| Both | 3088.1 (2721.0, 3508.7) | 3088.1 (2721.0, 3508.7) | 3088.1 (2721.0, 3508.7) | 197.0 (147.1, 247.5) | 73.6 (41.2, 117.3) |  |
| Female | 1386.3 (1216.1, 1575.9) | 1386.3 (1216.1, 1575.9) | 1386.3 (1216.1, 1575.9) | 83.3 (60.5, 107.2) | 30.9 (16.8, 49.9) |  |
| Male | 1701.8 (1478.3, 1954.8) | 1701.8 (1478.3, 1954.8) | 1701.8 (1478.3, 1954.8) | 113.6 (81.2, 150.3) | 42.6 (22.8, 69.8) |  |
| YLDs |  |  |  |  |  |  |
| Both | 1123.1 (873.3, 1403.9) | 1123.1 (873.3, 1403.9) | 1123.1 (873.3, 1403.9) | 0.3 (0.2, 0.4) | 0.1 (0.1, 0.2) |  |
| Female | 570.8 (444.5, 718.0) | 570.8 (444.5, 718.0) | 570.8 (444.5, 718.0) | 0.1 (0, 0.1) | 0.1 (0, 0.1) |  |
| Male | 552.2 (431.4, 692.0) | 552.2 (431.4, 692.0) | 552.2 (431.4, 692.0) | 0.1 (0.1, 0.2) | 0.1 (0, 0.1) |  |
| YLLs |  |  |  |  |  |  |
| Both | 1965.1 (1679.9, 2283.8) | 1965.1 (1679.9, 2283.8) | 1965.1 (1679.9, 2283.8) | 196.8 (146.9, 247.3) | 73.5 (41.2, 117.2) |  |
| Female | 815.5 (707.2, 933.6) | 815.5 (707.2, 933.6) | 815.5 (707.2, 933.6) | 83.3 (60.4, 107.1) | 30.9 (16.8, 49.8) |  |
| Male | 1149.6 (952.5, 1375.3) | 1149.6 (952.5, 1375.3) | 1149.6 (952.5, 1375.3) | 113.5 (81.1, 150.2) | 42.6 (22.8, 69.7) |  |
| **Attributable age-standardized rate per 100,000** | | | | | |  |
| Deaths |  |  |  |  |  |  |
| Both | 3.0 (2.6, 3.5) | 3.0 (2.6, 3.5) | 3.0 (2.6, 3.5) | 0.3 (0.2, 0.3) | 0.1 (0, 0.1) |  |
| Female | 2.7 (2.3, 3.1) | 2.7 (2.3, 3.1) | 2.7 (2.3, 3.1) | 0.2 (0.2, 0.3) | 0.1 (0, 0.1) |  |
| Male | 3.3 (2.7, 3.9) | 3.3 (2.7, 3.9) | 3.3 (2.7, 3.9) | 0.3 (0.2, 0.4) | 0.1 (0, 0.2) |  |
| DALYs |  |  |  |  |  |  |
| Both | 357.6 (313.9, 406.9) | 357.6 (313.9, 406.9) | 357.6 (313.9, 406.9) | 27.4 (20.5, 34.5) | 10.2 (5.7, 16.3) |  |
| Female | 333.0 (296.2, 374.9) | 333.0 (296.2, 374.9) | 333.0 (296.2, 374.9) | 25.2 (18.3, 32.4) | 9.3 (5.1, 15.0) |  |
| Male | 378.3 (326.6, 434.8) | 378.3 (326.6, 434.8) | 378.3 (326.6, 434.8) | 29.4 (21.0, 38.9) | 11 (5.9, 18.0) |  |
| YLDs |  |  |  |  |  |  |
| Both | 85.2 (66.4, 106.3) | 85.2 (66.4, 106.3) | 85.2 (66.4, 106.3) | 0 (0, 0.1) | 0 (0, 0) |  |
| Female | 87.8 (68.0, 109.8) | 87.8 (68.0, 109.8) | 87.8 (68.0, 109.8) | 0 (0, 0.1) | 0 (0, 0) |  |
| Male | 82.7 (64.2, 103.2) | 82.7 (64.2, 103.2) | 82.7 (64.2, 103.2) | 0 (0, 0.1) | 0 (0, 0) |  |
| YLLs |  |  |  |  |  |  |
| Both | 272.4 (232.9, 316.3) | 272.4 (232.9, 316.3) | 272.4 (232.9, 316.3) | 27.4 (20.4, 34.4) | 10.2 (5.7, 16.3) |  |
| Female | 245.2 (212.6, 280.6) | 245.2 (212.6, 280.6) | 245.2 (212.6, 280.6) | 25.1 (18.2, 32.3) | 9.3 (5.0, 15.0) |  |
| Male | 295.6 (245.0, 353.6) | 295.6 (245.0, 353.6) | 295.6 (245.0, 353.6) | 29.3 (20.9, 38.8) | 11.0 (5.8, 18) |  |

Abbreviation: DALYs, disability-adjusted life years; YLDs, years lived with disability; YLLs, years of life lost.

**Table S11** Points estimated and 95% uncertainty interval of attributable number and age-standardized rate of risk factors for neonatal encephalopathy due to birth asphyxia and trauma by gender in China, 2019

| **Measures** | **All risk factors** | **Low birth weight** | **Short gestation** | **Ambient particulate matter pollution** | **Household air pollution from solid fuels** |  |
| --- | --- | --- | --- | --- | --- | --- |
|  |  |  |  |  |  |  |
| **Attributable number in thousands** | | | | | |  |
| Deaths |  |  |  |  |  |  |
| Both | 9.1 (7.7, 10.9) | 6.8 (5.7, 8.1) | 5.6 (4.6, 6.6) | 1.4 (1.1, 1.8) | 0.5 (0.3, 0.9) |  |
| Female | 3.8 (3.2, 4.4) | 2.8 (2.4, 3.3) | 2.4 (2.0, 2.9) | 0.6 (0.4, 0.8) | 0.2 (0.1, 0.4) |  |
| Male | 5.3 (4.3, 6.5) | 3.9 (3.2, 4.8) | 3.1 (2.5, 3.9) | 0.8 (0.6, 1.1) | 0.3 (0.1, 0.5) |  |
| DALYs |  |  |  |  |  |  |
| Both | 817.3 (686.9, 971.3) | 609.2 (512.9, 726.0) | 500.6 (413.5, 593.7) | 131.9 (98.7, 168.6) | 53.0 (30.4, 83.5) |  |
| Female | 339.3 (285.4, 395.2) | 257.5 (214.8, 301.2) | 218.6 (181.6, 259.4) | 54.9 (39.5, 72.4) | 22.0 (12.2, 35.7) |  |
| Male | 477.9 (385.8, 583.6) | 351.6 (285.7, 433.3) | 282.0 (224.4, 352.6) | 76.9 (54.8, 105.8) | 30.9 (17.0, 49.9) |  |
| YLDs |  |  |  |  |  |  |
| Both | 0 (0, 0) | 0 (0, 0) | 0 (0, 0) | 0 (0, 0) | 0 (0, 0) |  |
| Female | 0 (0, 0) | 0 (0, 0) | 0 (0, 0) | 0 (0, 0) | 0 (0, 0) |  |
| Male | 0 (0, 0) | 0 (0, 0) | 0 (0, 0) | 0 (0, 0) | 0 (0, 0) |  |
| YLLs |  |  |  |  |  |  |
| Both | 817.3 (686.9, 971.3) | 609.2 (512.9, 726.0) | 500.6 (413.5, 593.7) | 131.9 (98.7, 168.6) | 53.0 (30.4, 83.5) |  |
| Female | 339.3 (285.4, 395.2) | 257.5 (214.8, 301.2) | 218.6 (181.6, 259.4) | 54.9 (39.5, 72.4) | 22.0 (12.2, 35.7) |  |
| Male | 477.9 (385.8, 583.6) | 351.6 (285.7, 433.3) | 282.0 (224.4, 352.6) | 76.9 (54.8, 105.8) | 30.9 (17.0, 49.9) |  |
| **Attributable age-standardized rate per 100,000** | | | | | |  |
| Deaths |  |  |  |  |  |  |
| Both | 1.2 (1.0, 1.5) | 0.9 (0.8, 1.1) | 0.7 (0.6, 0.9) | 0.2 (0.1, 0.2) | 0.1 (0, 0.1) |  |
| Female | 1.1 (0.9, 1.3) | 0.8 (0.7, 1.0) | 0.7 (0.6, 0.8) | 0.1 (0.1, 0.2) | 0.1 (0, 0.1) |  |
| Male | 1.3 (1.1, 1.7) | 1.0 (0.8, 1.2) | 0.8 (0.6, 1.0) | 0.2 (0.1, 0.3) | 0.1 (0, 0.1) |  |
| DALYs |  |  |  |  |  |  |
| Both | 114.0 (95.8, 135.5) | 84.9 (71.5, 101.2) | 69.8 (57.7, 82.8) | 18.4 (13.7, 23.5) | 7.4 (4.2, 11.6) |  |
| Female | 102.6 (86.3, 119.5) | 77.8 (64.9, 91.1) | 66.1 (54.9, 78.4) | 16.6 (11.9, 21.9) | 6.6 (3.7, 10.8) |  |
| Male | 123.7 (99.9, 151.1) | 91.0 (73.9, 112.2) | 73.0 (58.1, 91.3) | 19.9 (14.1, 27.4) | 8.0 (4.4, 12.9) |  |
| YLDs |  |  |  |  |  |  |
| Both | 0 (0, 0) | 0 (0, 0) | 0 (0, 0) | 0 (0, 0) | 0 (0, 0) |  |
| Female | 0 (0, 0) | 0 (0, 0) | 0 (0, 0) | 0 (0, 0) | 0 (0, 0) |  |
| Male | 0 (0, 0) | 0 (0, 0) | 0 (0, 0) | 0 (0, 0) | 0 (0, 0) |  |
| YLLs |  |  |  |  |  |  |
| Both | 114.0 (95.8, 135.5) | 84.9 (71.5, 101.2) | 69.8 (57.7, 82.8) | 18.4 (13.7, 23.5) | 7.4 (4.2, 11.6) |  |
| Female | 102.6 (86.3, 119.5) | 77.8 (64.9, 91.1) | 66.1 (54.9, 78.4) | 16.6 (11.9, 21.9) | 6.6 (3.7, 10.8) |  |
| Male | 123.7 (99.9, 151.1) | 91.0 (73.9, 112.2) | 73.0 (58.1, 91.3) | 19.9 (14.1, 27.3) | 8.0 (4.4, 12.9) |  |

Abbreviation: DALYs, disability-adjusted life years; YLDs, years lived with disability; YLLs, years of life lost.

**Table S12** Points estimated and 95% uncertainty interval of attributable number and age-standardized rate of risk factors for neonatal sepsis and other neonatal infections by gender in China, 2019

| **Measures** | **All risk factors** | **Low birth weight** | **Short gestation** | **Ambient particulate matter pollution** | **Household air pollution from solid fuels** |  |
| --- | --- | --- | --- | --- | --- | --- |
|  |  |  |  |  |  |  |
| **Attributable number in thousands** | | | | | |  |
| Deaths |  |  |  |  |  |  |
| Both | 0.9 (0.8, 1.1) | 0.7 (0.6, 0.9) | 0.5 (0.4, 0.6) | 0.2 (0.1, 0.2) | 0.1 (0, 0.1) |  |
| Female | 0.3 (0.3, 0.4) | 0.3 (0.2, 0.3) | 0.2 (0.1, 0.2) | 0.1 (0, 0.1) | 0 (0, 0) |  |
| Male | 0.5 (0.4, 0.7) | 0.4 (0.3, 0.5) | 0.3 (0.2, 0.4) | 0.1 (0.1, 0.1) | 0 (0, 0.1) |  |
| DALYs |  |  |  |  |  |  |
| Both | 86.9 (72.4, 103.2) | 67.5 (56.0, 80.4) | 51.8 (42.6, 61.9) | 14.8 (11.2, 18.8) | 5.0 (2.7, 8.2) |  |
| Female | 34.2 (29.0, 40.4) | 27.0 (22.8, 31.8) | 21.2 (17.7, 25.3) | 5.9 (4.4, 7.6) | 1.9 (1.0, 3.1) |  |
| Male | 52.6 (41.5, 64.2) | 40.4 (31.6, 49.5) | 30.5 (23.9, 37.4) | 8.9 (6.4, 11.7) | 3.1 (1.6, 5.0) |  |
| YLDs |  |  |  |  |  |  |
| Both | 0.2 (0.1, 0.3) | 0 (0, 0) | 0 (0, 0) | 0.2 (0.1, 0.3) | 0.1 (0, 0.1) |  |
| Female | 0.1 (0.1, 0.2) | 0 (0, 0) | 0 (0, 0) | 0.1 (0.1, 0.1) | 0 (0, 0) |  |
| Male | 0.1 (0.1, 0.2) | 0 (0, 0) | 0 (0, 0) | 0.1 (0.1, 0.1) | 0 (0, 0.1) |  |
| YLLs |  |  |  |  |  |  |
| Both | 86.7 (72.2, 103.0) | 67.5 (56.0, 80.4) | 51.8 (42.6, 61.9) | 14.6 (11.0, 18.6) | 5.0 (2.7, 8.1) |  |
| Female | 34.1 (29.0, 40.3) | 27.0 (22.8, 31.8) | 21.2 (17.7, 25.3) | 5.9 (4.4, 7.6) | 1.9 (1.0, 3.1) |  |
| Male | 52.5 (41.4, 64.1) | 40.4 (31.6, 49.5) | 30.5 (23.9, 37.4) | 8.8 (6.3, 11.6) | 3.0 (1.6, 5.0) |  |
| **Attributable age-standardized rate per 100,000** | | | | | |  |
| Deaths |  |  |  |  |  |  |
| Both | 0.1 (0.1, 0.2) | 0.1 (0.1, 0.1) | 0.1 (0.1, 0.1) | 0 (0, 0) | 0 (0, 0) |  |
| Female | 0.1 (0, 0.1) | 0.1 (0.1, 0.1) | 0.1 (0.1, 0.1) | 0 (0, 0) | 0 (0, 0) |  |
| Male | 0.1 (0.1, 0.2) | 0.1 (0.1, 0.1) | 0.1 (0.1, 0.1) | 0 (0, 0) | 0 (0, 0) |  |
| DALYs |  |  |  |  |  |  |
| Both | 12.1 (10.0, 14.3) | 9.3 (7.8, 11.1) | 7.2 (5.9, 8.6) | 2.0 (1.5, 2.6) | 0.7 (0.3, 1.1) |  |
| Female | 10.3 (8.7, 12.2) | 8.1 (6.8, 9.6) | 6.4 (5.3, 7.6) | 1.7 (1.3, 2.3) | 0.5 (0.3, 0.9) |  |
| Male | 13.6 (10.7, 16.6) | 10.4 (8.1, 12.7) | 7.8 (6.1, 9.6) | 2.2 (1.6, 3.0) | 0.8 (0.4, 1.3) |  |
| YLDs |  |  |  |  |  |  |
| Both | 0 (0, 0) | 0 (0, 0) | 0 (0, 0) | 0 (0, 0) | 0 (0, 0) |  |
| Female | 0 (0, 0.1) | 0 (0, 0) | 0 (0, 0) | 0 (0, 0) | 0 (0, 0) |  |
| Male | 0 (0, 0) | 0 (0, 0) | 0 (0, 0) | 0 (0, 0) | 0 (0, 0) |  |
| YLLs |  |  |  |  |  |  |
| Both | 12.0 (10.0, 14.3) | 9.3 (7.8, 11.1) | 7.2 (5.9, 8.6) | 2.0 (1.5, 2.5) | 0.6 (0.3, 1.1) |  |
| Female | 10.3 (8.7, 12.1) | 8.1 (6.8, 9.6) | 6.4 (5.3, 7.6) | 1.7 (1.3, 2.2) | 0.5 (0.3, 0.9) |  |
| Male | 13.5 (10.7, 16.5) | 10.4 (8.1, 12.7) | 7.8 (6.1, 9.6) | 2.2 (1.6, 3.0) | 0.7 (0.4, 1.2) |  |

Abbreviation: DALYs, disability-adjusted life years; YLDs, years lived with disability; YLLs, years of life lost.

**Table S13** Points estimated and 95% uncertainty interval of attributable number and age-standardized rate of risk factors for hemolytic disease and other neonatal jaundice by gender in China, 2019

| **Measures** | **All risk factors** | **Low birth weight** | **Short gestation** | **Ambient particulate matter pollution** | **Household air pollution from solid fuels** |  |
| --- | --- | --- | --- | --- | --- | --- |
|  |  |  |  |  |  |  |
| **Attributable number in thousands** | | | | | |  |
| Deaths |  |  |  |  |  |  |
| Both | 0.4 (0.3, 0.5) | 0.3 (0.2, 0.3) | 0.2 (0.2, 0.3) | 0.1 (0, 0.1) | 0 (0, 0) |  |
| Female | 0.1 (0.1, 0.1) | 0.1 (0.1, 0.1) | 0.1 (0.1, 0.1) | 0 (0, 0) | 0 (0, 0) |  |
| Male | 0.2 (0.2, 0.3) | 0.2 (0.1, 0.2) | 0.2 (0.1, 0.2) | 0 (0, 0.1) | 0 (0, 0) |  |
| DALYs |  |  |  |  |  |  |
| Both | 37.4 (31.1, 45.0) | 29.2 (24.1, 35.0) | 22.0 (18.1, 26.4) | 5.7 (4.2, 7.5) | 2.6 (1.6, 4.0) |  |
| Female | 14.0 (11.7, 16.7) | 11.1 (9.3, 13.1) | 8.6 (7.2, 10.2) | 2.2 (1.5, 2.8) | 0.9 (0.5, 1.5) |  |
| Male | 23.4 (18.7, 28.9) | 18.1 (14.5, 22.2) | 13.3 (10.6, 16.6) | 3.5 (2.5, 4.8) | 1.6 (0.9, 2.6) |  |
| YLDs |  |  |  |  |  |  |
| Both | 0 (0, 0.1) | 0 (0, 0) | 0 (0, 0) | 0 (0, 0.1) | 0 (0, 0) |  |
| Female | 0 (0, 0) | 0 (0, 0) | 0 (0, 0) | 0 (0, 0) | 0 (0, 0) |  |
| Male | 0 (0, 0.1) | 0 (0, 0) | 0 (0, 0) | 0 (0, 0) | 0 (0, 0) |  |
| YLLs |  |  |  |  |  |  |
| Both | 37.4 (31.1, 45.0) | 29.2 (24.1, 35.0) | 22.0 (18.1, 26.4) | 5.7 (4.2, 7.5) | 2.6 (1.5, 4.0) |  |
| Female | 14.0 (11.7, 16.6) | 11.1 (9.3, 13.1) | 8.6 (7.2, 10.2) | 2.2 (1.5, 2.8) | 0.9 (0.5, 1.5) |  |
| Male | 23.3 (18.6, 28.9) | 18.1 (14.5, 22.2) | 13.3 (10.6, 16.6) | 3.5 (2.5, 4.8) | 1.6 (0.9, 2.6) |  |
| **Attributable age-standardized rate per 100,000** | | | | | |  |
| Deaths |  |  |  |  |  |  |
| Both | 0.1 (0, 0.1) | 0 (0, 0.1) | 0 (0, 0) | 0 (0, 0) | 0 (0, 0) |  |
| Female | 0 (0, 0.1) | 0 (0, 0) | 0 (0, 0) | 0 (0, 0) | 0 (0, 0) |  |
| Male | 0.1 (0.1, 0.1) | 0.1 (0, 0.1) | 0 (0, 0) | 0 (0, 0) | 0 (0, 0) |  |
| DALYs |  |  |  |  |  |  |
| Both | 5.2 (4.3, 6.2) | 4.0 (3.3, 4.8) | 3.0 (2.5, 3.6) | 0.8 (0.5, 1.0) | 0.3 (0.2, 0.5) |  |
| Female | 4.2 (3.5, 5.0) | 3.3 (2.8, 3.9) | 2.6 (2.1, 3.0) | 0.6 (0.4, 0.8) | 0.3 (0.1, 0.4) |  |
| Male | 6.0 (4.8, 7.4) | 4.6 (3.7, 5.7) | 3.4 (2.7, 4.2) | 0.9 (0.6, 1.2) | 0.4 (0.2, 0.6) |  |
| YLDs |  |  |  |  |  |  |
| Both | 0 (0, 0) | 0 (0, 0) | 0 (0, 0) | 0 (0, 0) | 0 (0, 0) |  |
| Female | 0 (0, 0) | 0 (0, 0) | 0 (0, 0) | 0 (0, 0) | 0 (0, 0) |  |
| Male | 0 (0, 0) | 0 (0, 0) | 0 (0, 0) | 0 (0, 0) | 0 (0, 0) |  |
| YLLs |  |  |  |  |  |  |
| Both | 5.2 (4.3, 6.2) | 4.0 (3.3, 4.8) | 3.0 (2.5, 3.6) | 0.8 (0.5, 1.0) | 0.3 (0.2, 0.5) |  |
| Female | 4.2 (3.5, 5.0) | 3.3 (2.8, 3.9) | 2.6 (2.1, 3.0) | 0.6 (0.4, 0.8) | 0.2 (0.1, 0.4) |  |
| Male | 6.0 (4.8, 7.4) | 4.6 (3.7, 5.7) | 3.4 (2.7, 4.2) | 0.9 (0.6, 1.2) | 0.4 (0.2, 0.6) |  |

Abbreviation: DALYs, disability-adjusted life years; YLDs, years lived with disability; YLLs, years of life lost.


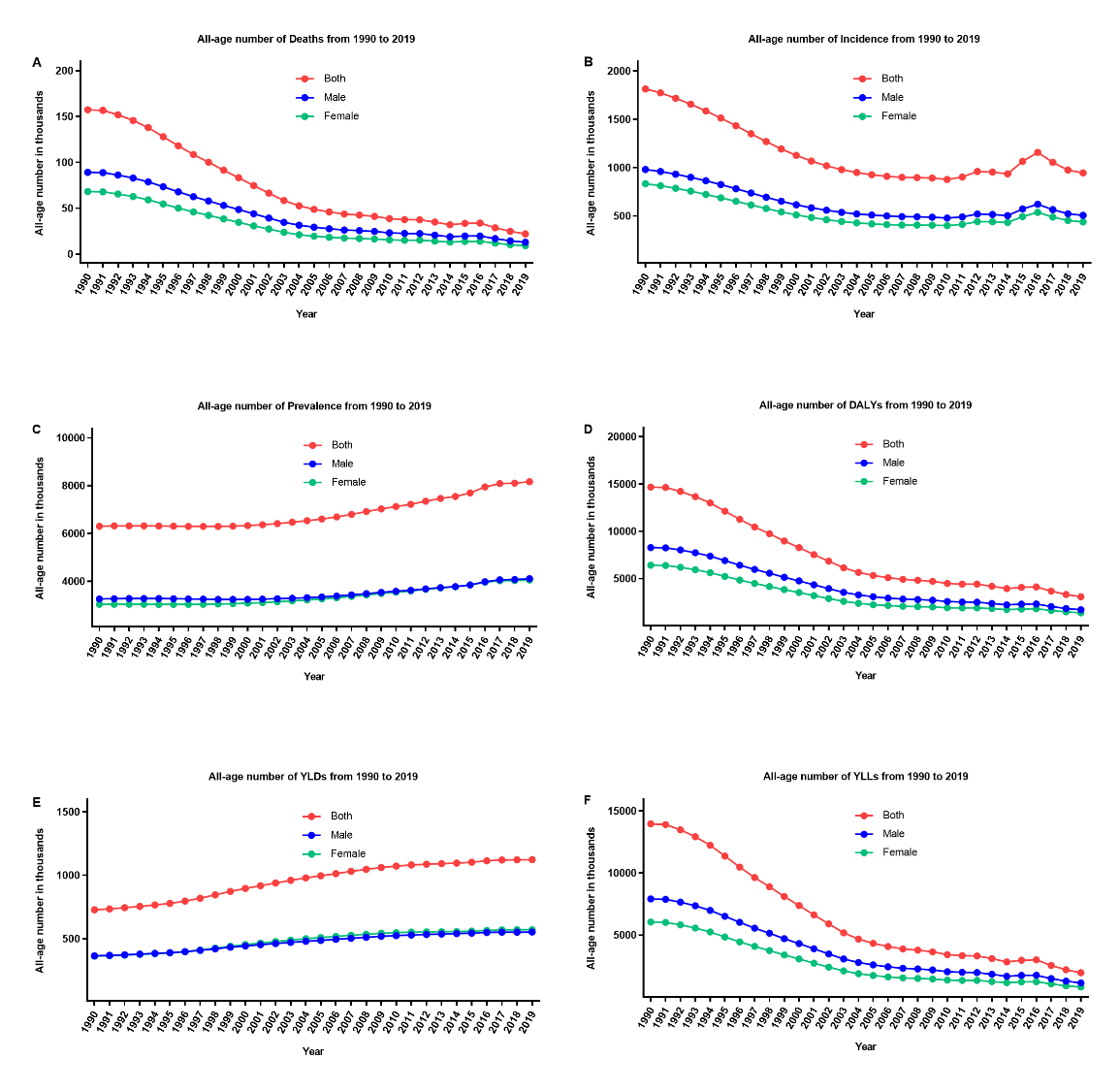


(A) Deaths; (B) incidence; (C) prevalence; (D) DALYs; (E) YLDs; (F) YLLs.

**Figure S1** The trend of all-age number of all six measures for neonatal preterm birth in China from 1990 to 2019


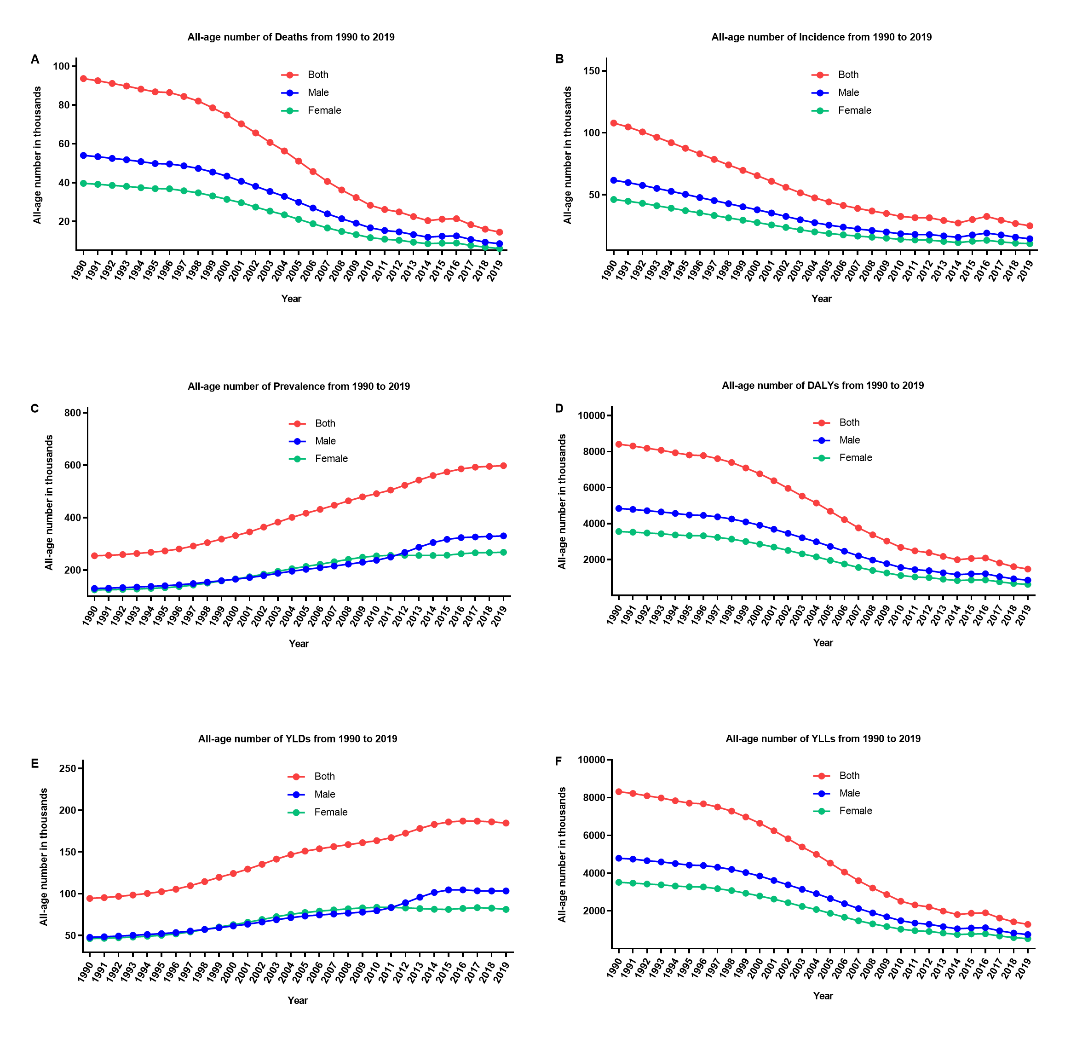


(A) Deaths; (B) incidence; (C) prevalence; (D) DALYs; (E) YLDs; (F) YLLs.

**Figure S2** The trend of all-age number of all six measures for neonatal encephalopathy due to birth asphyxia and trauma in China from 1990 to 2019


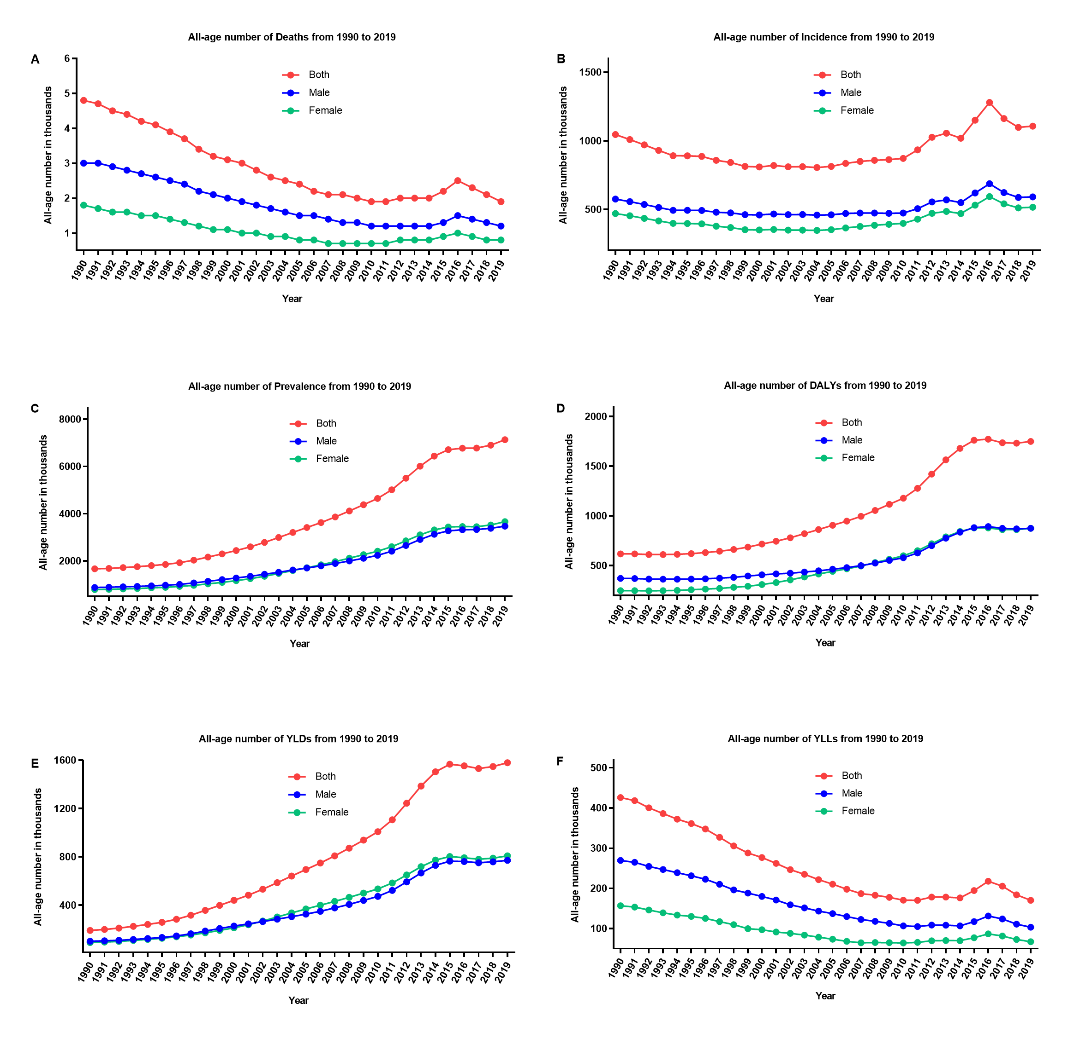


(A) Deaths; (B) incidence; (C) prevalence; (D) DALYs; (E) YLDs; (F) YLLs.

**Figure S3** The trend of all-age number of all six measures for neonatal sepsis and other neonatal infections in China from 1990 to 2019


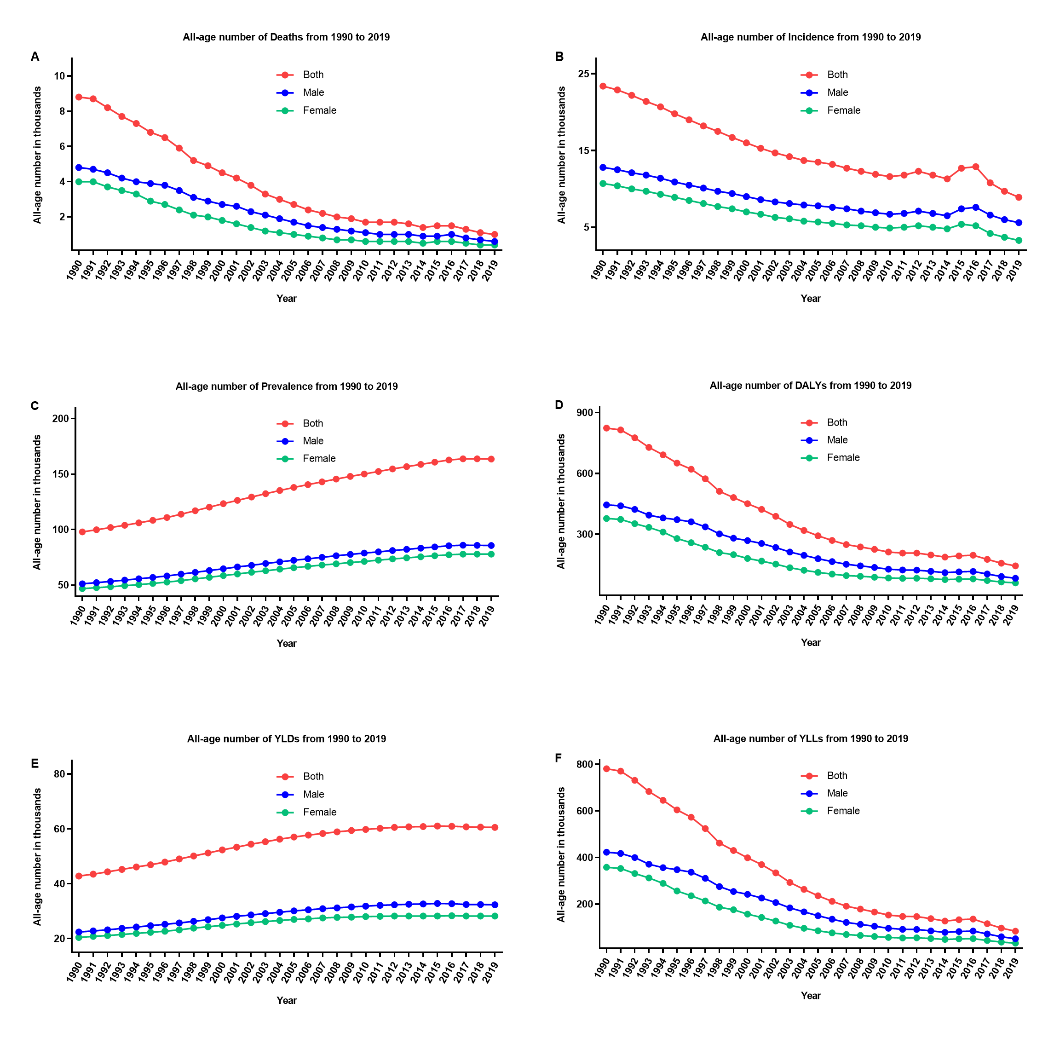


(A) Deaths; (B) incidence; (C) prevalence; (D) DALYs; (E) YLDs; (F) YLLs.

**Figure S4** The trend of all-age number of all six measures for hemolytic disease and other neonatal jaundice in China from 1990 to 2019


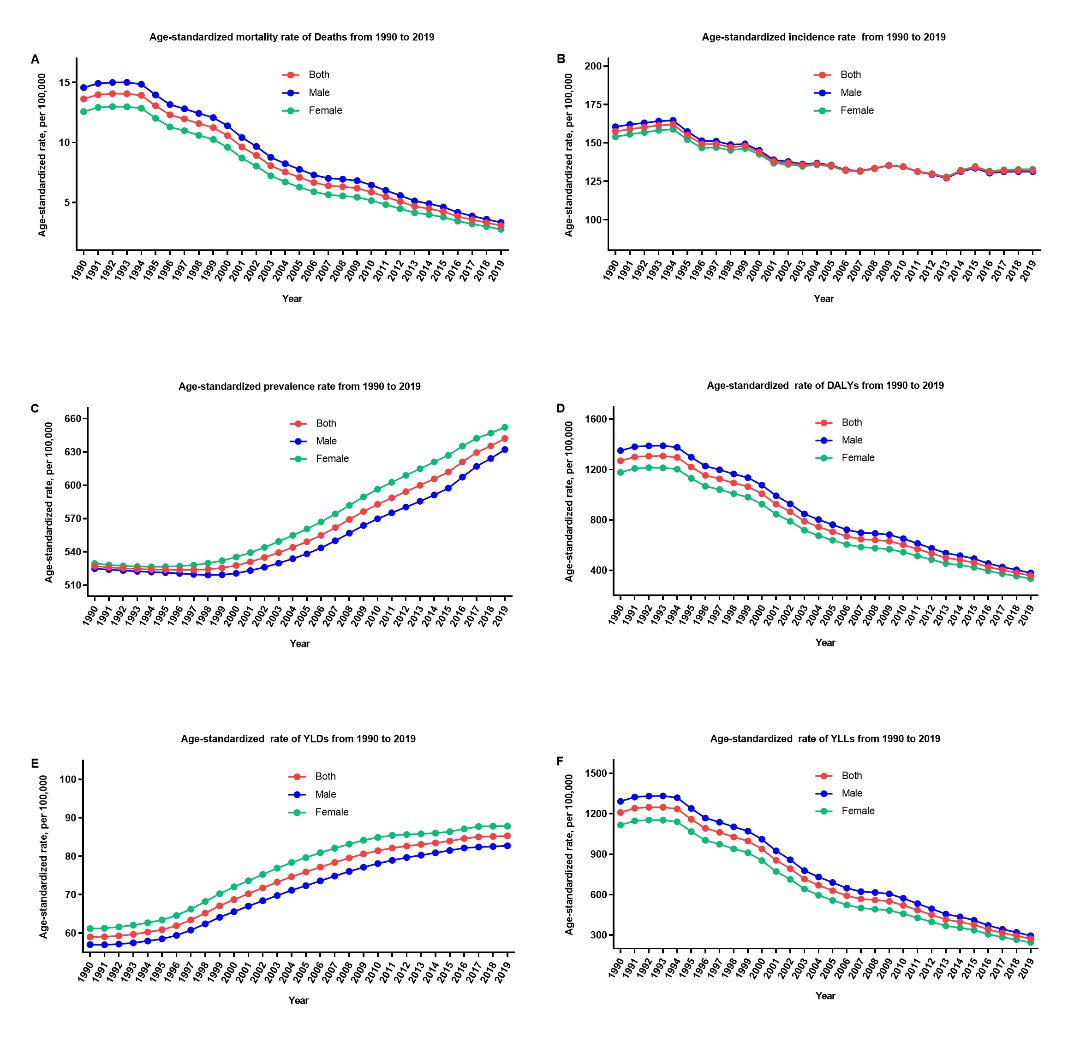


(A) Mortality rate; (B) incidence rate; (C) prevalence rate; (D) DALYs rate; (E) YLDs rate; (F) YLLs rate.

**Figure S5** The trend of age-standardized rate of all six measures for neonatal preterm birth in China from 1990 to 2019


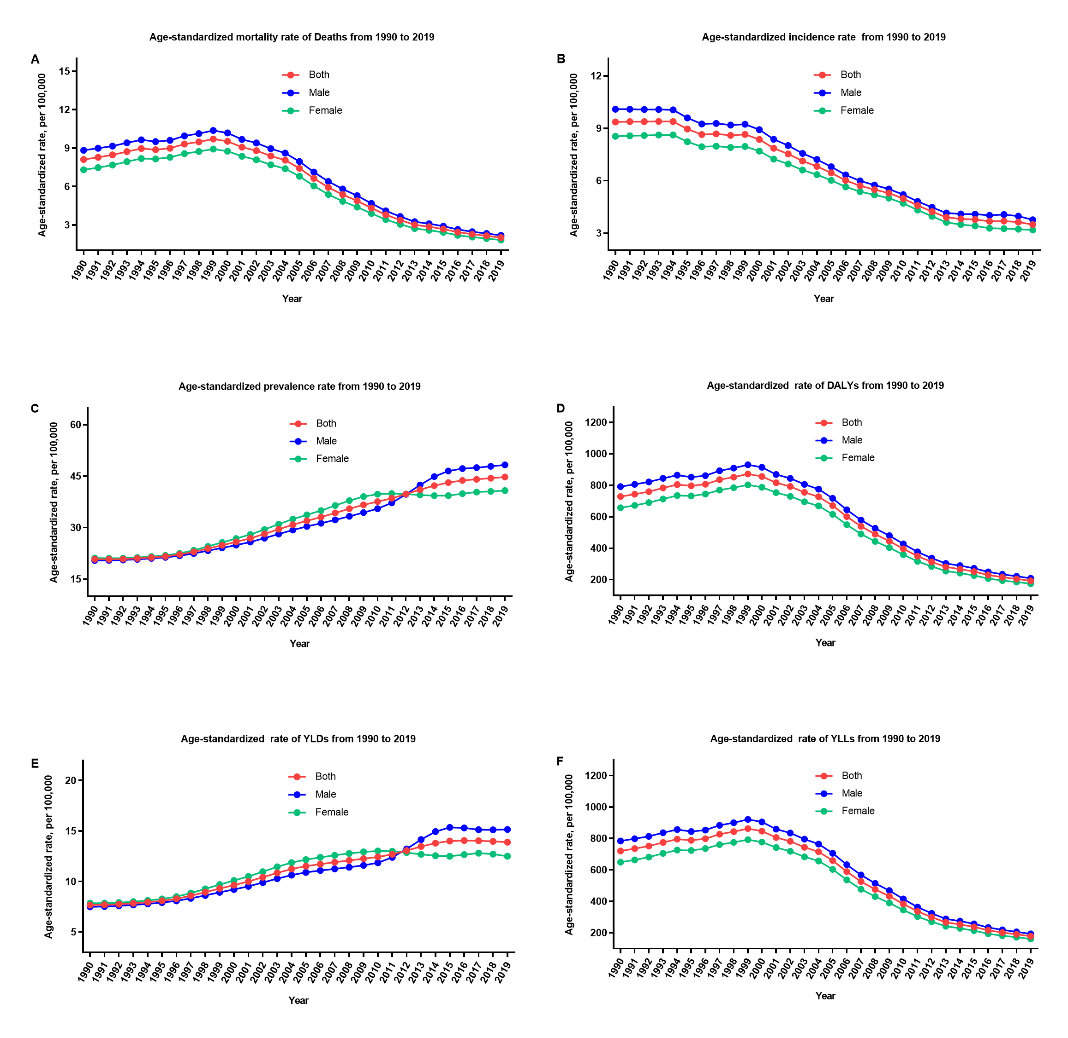


(A) Mortality rate; (B) incidence rate; (C) prevalence rate; (D) DALYs rate; (E) YLDs rate; (F) YLLs rate.

**Figure S6** The trend of age-standardized rate of all six measures for neonatal encephalopathy due to birth asphyxia and trauma in China from 1990 to 2019


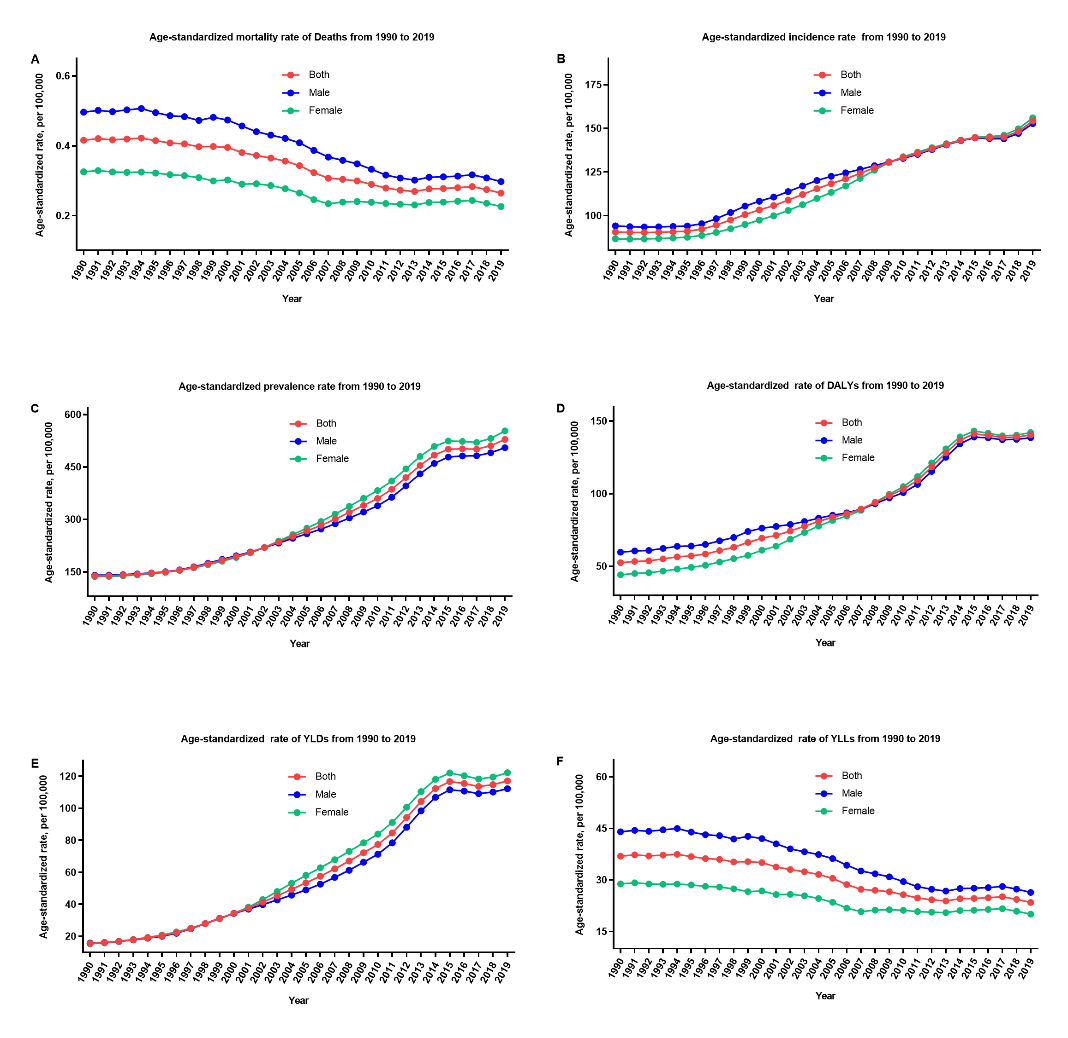


(A) Mortality rate; (B) incidence rate; (C) prevalence rate; (D) DALYs rate; (E) YLDs rate; (F) YLLs rate.

**Figure S7** The trend of age-standardized rate of all six measures for neonatal sepsis and other neonatal infections in China from 1990 to 2019


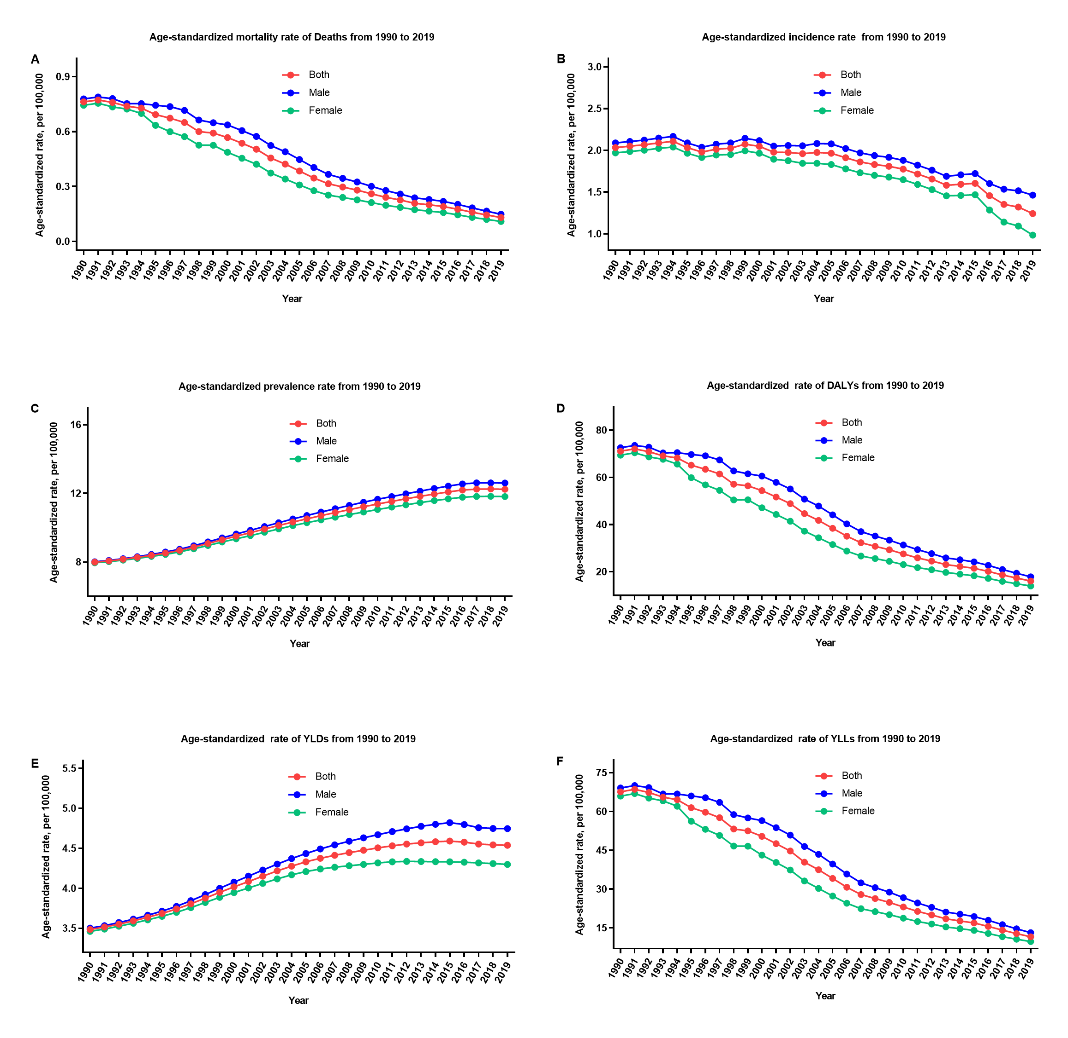


(A) Mortality rate; (B) incidence rate; (C) prevalence rate; (D) DALYs rate; (E) YLDs rate; (F) YLLs rate.

**Figure S8** The trend of age-standardized rate of all six measures for hemolytic disease and other neonatal jaundice in China from 1990 to 2019


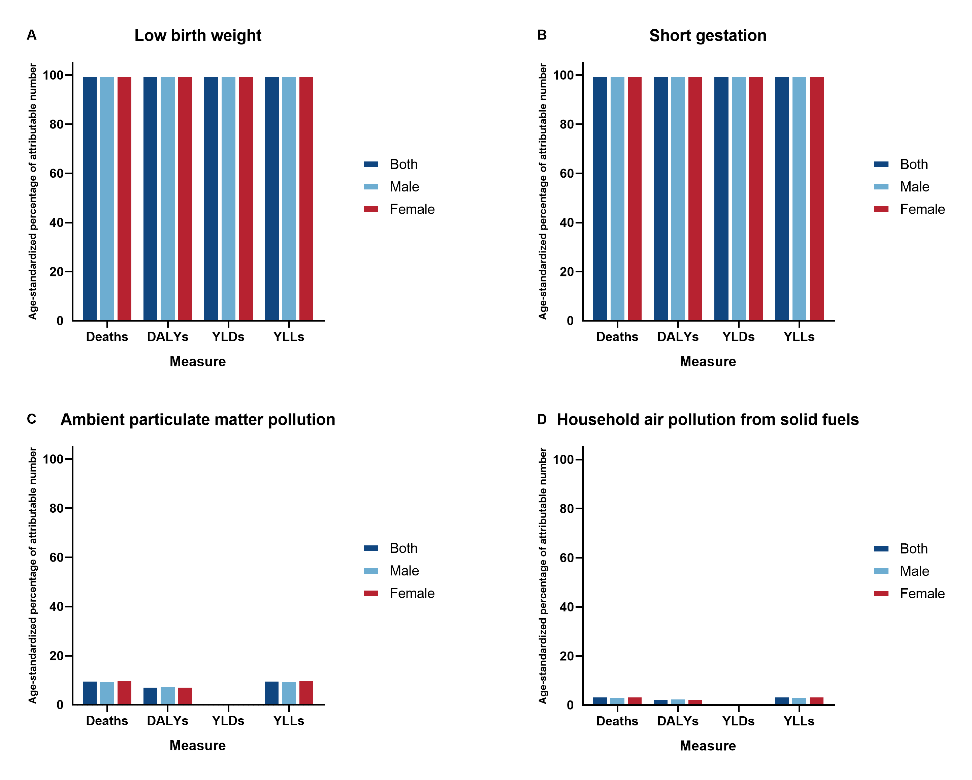


(A) low birth weight; (B) short gestation; (C) ambient particulate matter pollution; (D) Household air pollution from solid fuels.

**Figure S9** Age-standardized percentage of attributable number of risk factors of four measures for neonatal preterm birth by gender in China, 2019


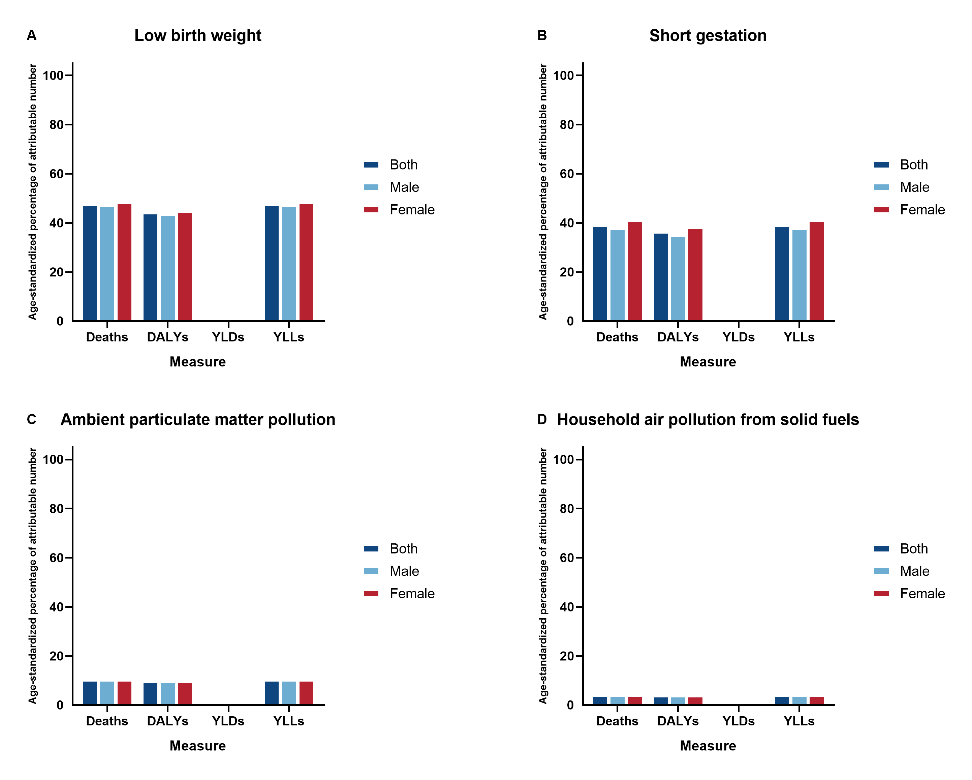


(A) low birth weight; (B) short gestation; (C) ambient particulate matter pollution; (D) Household air pollution from solid fuels.

**Figure S10** Age-standardized percentage of attributable number of risk factors of four measures for neonatal encephalopathy due to birth asphyxia and trauma by gender in China, 2019


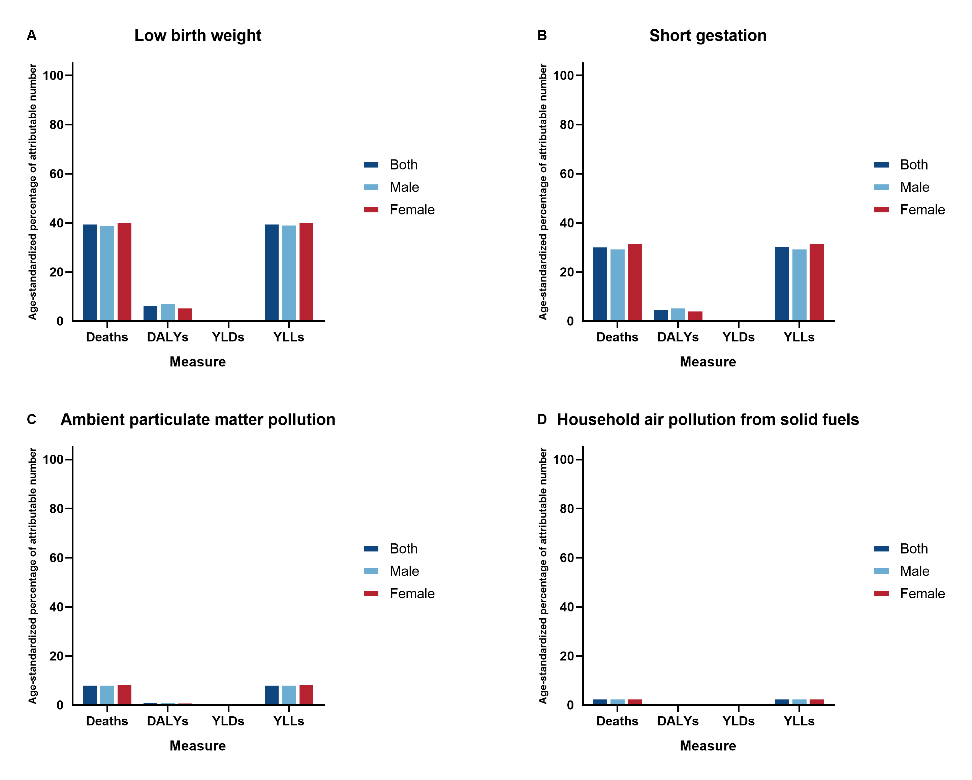


(A) low birth weight; (B) short gestation; (C) ambient particulate matter pollution; (D) Household air pollution from solid fuels.

**Figure S11** Age-standardized percentage of attributable number of risk factors of four measures for neonatal sepsis and other neonatal infections by gender in China, 2019


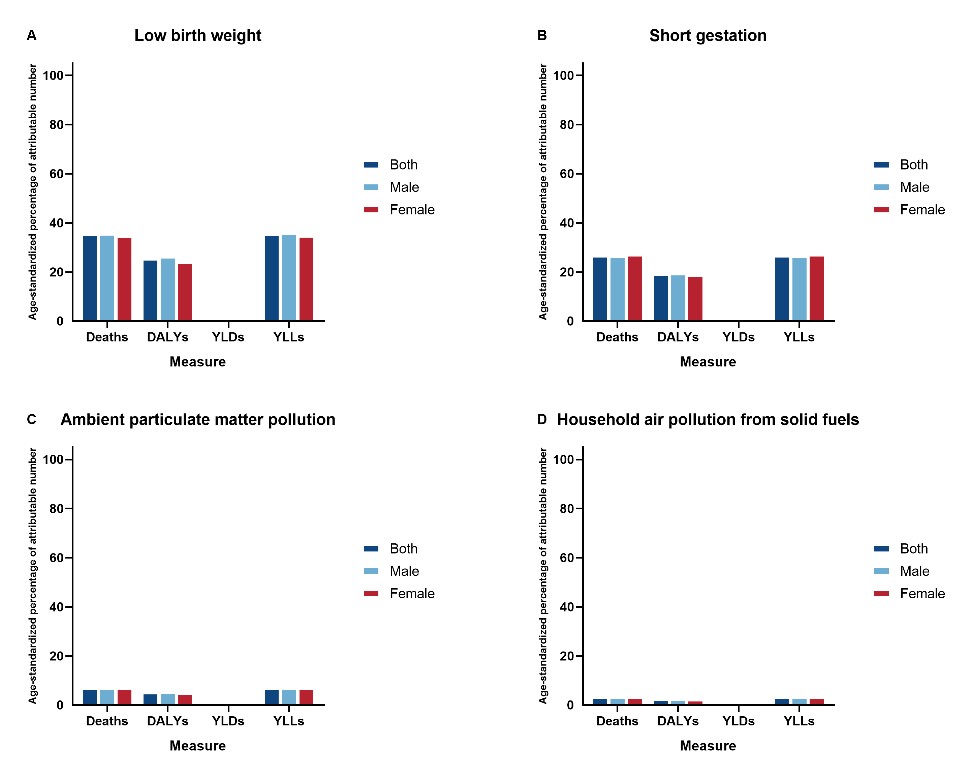


(A) low birth weight; (B) short gestation; (C) ambient particulate matter pollution; (D) Household air pollution from solid fuels.

**Figure S12** Age-standardized percentage of attributable number of risk factors of four measures for hemolytic disease and other neonatal jaundice by gender in China, 2019
